# Identification and characterization of *CmPP2C31* playing a positive role in the abiotic stress resistance of Chinese chestnut via an integrated strategy

**DOI:** 10.3389/fpls.2024.1491269

**Published:** 2024-12-13

**Authors:** Xuan Wang, Wenli Shang, Mingyuan Li, Fei Cao, Dongsheng Wang, Meng Wang, Yi Lu, Haie Zhang, Fei Shen, Jing Liu

**Affiliations:** ^1^ Engineering Research Center of Chestnut Industry Technology, Ministry of Education, Hebei Normal University of Science and Technology, Qinhuangdao, Hebei, China; ^2^ Hebei Key Laboratory of Horticultural Germplasm Excavation and Innovative Utilization, College of Horticulture Science and Technology, Hebei Normal University of Science and Technology, Changli, Hebei, China; ^3^ Rural Revitalization Research Center, Hebei Normal University of Science and Technology, Qinhuangdao, Hebei, China; ^4^ Institute of Biotechnology, Beijing Academy of Agriculture and Forestry Sciences, Beijing, China

**Keywords:** Chinese chestnut, protein phosphatase 2C, abiotic stress, pollen magnetofection, yeast one-hybrid, *CmPP2C31*, RNA-seq

## Abstract

Chinese chestnut (*Castanea mollissima* Blume) is an important economic forest tree species and mainly cultivated in mountainous areas and wastelands, subjecting it to various abiotic stresses. The protein phosphatase 2C (PP2C) genes contributes largely to stress responses in plants. However, the characteristics and functions of *PP2C* genes in *C. mollissima* remain unknown. This study provides comprehensive analyses (including phylogenetic, synteny, RNA-seq, transgenic and yeast one-hybrid methods) revealing the characteristics of *CmPP2C* gene, which plays an important role in response to abiotic stress. Here, we identified 68 *CmPP2Cs* in the Chinese chestnut genome, and analyzed their characteristics and phylogenetic relationships. Furthermore, synteny analysis revealed that segmental and tandem duplication drove the expansion of the *CmPP2C* family to adapt to natural environmental pressures. RNA sequencing and co-expression analyses indicated that four hub *CmPP2Cs* in two key modules probably play important roles in the resistance to abiotic stress in chestnut. Among them, *CmPP2C31* was significantly down-regulated under drought stress. Transgenic experiments via pollen magnetofection revealed that *CmPP2C31* could positively and significantly regulate the drought resistance of Chinese chestnut seedlings. Subcellular localization showed that CmPP2C31 was a nuclear protein. Yeast one-hybrid assays suggested that EVM0007407 could regulate *CmPP2C31* expression by binding to its promoter, thereby participating in abiotic stress resistance. These findings in our study provided detailed information on the *CmPP2C* family genes and laid a foundation for further elucidating the molecular mechanism of resistance to abiotic stress chestnut.

## 1 Introduction

The Chinese chestnut (*Castanea mollissima* Blume) is economically important for nut and timber production, besides its ecological and social value. In China, the *C. mollissima* has the highest nut productivity and quality in the Fagaceae family, and contributes to poverty eradication among farmers (Ji et al., 2018). However, the crop is mainly cultivated in barren, arid mountainous areas and sandy wastelands, subjecting it to various abiotic stresses, including drought, cold, and high temperatures (Zhang et al., 2024). These abiotic stresses can severely impact growth, development, and yield of Chinese chestnut trees. Thus, identifying major stress resistance genes in Chinese chestnut and clarifying the gene regulation mechanisms is key for germplasm resource identification and strategic guidance of Chinese chestnut cultivation and management programs.

Reversible phosphorylation is an important protein modification process and involved in a variety of developmental processes and stress responses in plants (Zhang et al., 2013; Wu et al., 2022). Protein phosphorylation and dephosphorylation are catalyzed by protein kinases (PKs) and protein phosphatases (PPs), respectively (Pan et al., 2024). PPs in plants can be divided into protein tyrosine phosphatases (PTP) and protein serine/threonine phosphatases (PSP) based on their substrate specificity. Further, PSP includes two families: phosphoprotein phosphatase (PPP) and phosphoprotein metallo phosphatases (PPM) (Hu and Li, 2007; Pan et al., 2024). There are significant differences in amino acid sequences and advanced structures between members of PPP (PP1, PP2A, PP2B, PP4, PP5, PP6, and PP7) and PPM (PP2C and pyruvate dehydrogenase phosphatase) families (Cohen, 1989; Kerk et al., 2008; Bhalothia et al., 2016; Fan et al., 2019; Chen et al., 2022). Among them, PP2C is widely present in higher plants and contains a conserved catalytic domain on its C-termini. Unlike PPP family members, the catalytic function of PP2C depends on Mg^2+^ or Mn^2+^, due to the lack of regulatory subunits (Cohen, 1989; Kamada et al., 2020).

Extensive research has demonstrated that PP2C are important protein phosphatases involved in plant responses to a variety of abiotic stresses, including drought, high temperature, low temperature, and salinity (Sun et al., 2011; Wang et al., 2021; Chen et al., 2022; Pan et al., 2024). Firstly, PP2C plays a central role in the perception and transduction of the abscisic acid (ABA) signal. ABA is a key regulatory factor in plant responses to abiotic stresses, and PP2C acts as a negative regulator of the ABA signaling pathway. In maize, ZmPP2C-A10 interacts with ZmSnRK2 and ZmPYL (ABA receptors) to function as a negative regulator of drought tolerance (Xiang et al., 2017). *PP2C* genes, in pear and wheat, are generally sensitive to stress, *PbPP2C1* and *TaPP2C-a10* negatively regulates abiotic stresses responses via ABA signaling (Yu et al., 2019, 2020; Wang et al., 2021). Secondly, PP2Cs also participate in the regulation of other signaling pathways involved in plant responses to abiotic stresses (Soon et al., 2012; Jung et al., 2020; Lu et al., 2020). Studies have found that PP2C is crucial in mitogen-activated protein kinases (MAPKs) and ABA signaling (Schweighofer et al., 2007; Umezawa et al., 2009). PP2C49 negatively regulated AtHKT1 and further influenced Na^+^ distribution and extrusion under salt stress (Chu et al., 2021). Furthermore, PP2Cs can also regulate the calcium signaling pathway, which is involved in plant responses to low-temperature stress (Kudla et al., 2010). In addition, the expression of *PP2C* genes themselves is also regulated by stress factors. Studies have shown that the expression levels of multiple *PP2C* genes are significantly upregulated under drought, high temperature, and salt stress conditions, and these *PP2C* genes are involved in the plant’s response to the corresponding stresses (Bhaskara et al., 2012; Chen et al., 2022; Pan et al., 2024). Overall, the *PP2C* gene family plays a crucial role in plant responses to abiotic stresses. Further research on the regulatory mechanisms of *PP2C* genes will help us to deepen our understanding of the molecular mechanisms of plant stress tolerance and provide a theoretical basis for breeding stress-tolerant crop varieties.

The *PP2C* genes have been identified and functionally studied in multiple species. For example, 80 and 78 members of *PP2C* gene family have been predicted in *Arabidopsis thaliana* and rice (*Oryza sativa*), respectively (Xue et al., 2008). Eighteen *PP2C* genes were identified in soybean (*Glycine max*) (Shen et al., 2022), 92 in tomato (*Solanum lycopersicum*) (Qiu et al., 2022), 78 in potato (*Solanum tuberosum*) (Wang Y. et al., 2020), 128 in apple (*Malus domestica*), 118 in Chinese white pear (*Pyrus bretschneideri*) (Wang et al., 2021), 41 in walnut (*Juglans regia*) (Chen et al., 2022), and 60 in jute (*Corchorus capsularis*) (Pan et al., 2024). Further, all the above studies indicated that the *PP2C* genes play an important role in plant response to biotic/abiotic stresses. However, there are no reports on the identification and functionality of *PP2C* genes in Chinese chestnut, which limits the research on the stress regulatory mechanism and strategic application of *PP2C*s for Chinese chestnut development.

The aim of this study was to identify *PP2C* gene from Chinese chestnut genome, which plays an important role in response to abiotic stress. Synteny analysis revealed that tandem and segmental duplication drove the expansion of the *CmPP2C* family to cope with stress. RNA sequencing and co-expression analyses indicated that four hub *CmPP2C*s (especially *CmPP2C31*) in two key modules probably play important roles in the response to abiotic stress in Chinese chestnut. Furthermore, this study used subcellular localization, transgenic experiment, drought treatment, and yeast one hybridization (YIH) to reveal the protein function and regulatory relationship of CmPP2C31 and its upstream regulator. CmPP2C31 is a nucleus protein. The expression of *CmPP2C31* gene was regulated by the transcription factor of EVM0007407. Over-expressing *CmPP2C31* could significantly enhance drought resistance in Chinese chestnut seedlings. These findings provide comprehensive information and novel insights into the functions and regulatory mechanisms of Chinese chestnut *PP2C*s, laying a foundation for further molecular characterization of resistance to abiotic stress in Chinese chestnut.

## 2 Materials and methods

### 2.1 Identification and characterization of *CmPP2C* genes

The hidden Markov model profile of PP2C (PF00481) was used to search for *PP2C* genes in the N11-1 genome of Chinese chestnut (Wang J. et al., 2020). The results were validated by Pfam (http://pfam.xfam.org/search) and NCBI Batch CD-Search with the CDD database (https://www.ncbi.nlm.nih.gov/Structure/bwrpsb/bwrpsb.cgi). The CmPP2C amino acid sequences were analyzed on ExPASy (https://www.expasy.org) for length, isoelectric point, and relative molecular weight.

We performed multiple PP2C full-length sequence alignments and constructed phylogenetic tree using MEGA X (Kumar et al., 2018). Clustal W was used to create multiple sequence alignments. A neighbor-joining phylogenetic tree was constructed using p-distance substitution model and partial deletion gaps data treatment, and node support was estimated by conducting 1000 bootstrap replicates.

Conserved motifs in CmPP2Cs were analyzed using the MEME web server (http://meme-suite.org/) with maximum motif number set to 20. The exon-intron organization of *CmPP2C*s was analyzed using general feature format (GFF3) files and visualized on the Gene Structure Display Server (http://gsds.cbi.pku.edu.cn/). The *cis*-acting elements in gene promoters were identified by PlantCARE (https://bioinformatics.psb.ugent.be/webtools/plantcare/html/) using 1,500 bp upstream sequence of the transcription start site of each gene.

### 2.2 Chromosomal localization and synteny analyses

We constructed the chromosomal localization map of *CmPP2C*s using Mapchart 2.32 software (Voorrips, 2002). The syntenic gene pairs within the chestnut genome were identified by MCScanX, and displayed using Circos (version 0.69-8) software. The substitution rates of nonsynonymous (Ka) and synonymous (Ks) were calculated using the KaKs-calculator (version 2.0) with default genetic code table (Standard Code) and default method for estimating Ka and Ks and theirs references (Model Averaging on a set of candidate models) (Wang et al., 2010). The analysis of synteny between the genomes was performed by the Python version MCscan in JCVI utility libraries v1.0.5 (Tang et al., 2024).

### 2.3 Plant materials for RNA-seq and abiotic stress treatments

The plant materials used in this study were one-year-old seedlings of ‘Yanbao’ Chinese chestnut (a widely planted cultivar), which were planted in the greenhouse (temperature: 24 ± 2 °C; air humidity: 75 ± 5%; day-night rhythm: 14h light/10h dark) of the Hebei Normal University of Science and Technology. All seedlings were planted in pots 40 cm high and 38 cm in diameter, with a mixed substrate of grass charcoal:perlite:vermiculite ratio of 3:1:1. These seedlings were subjected to low temperature (cold, CD), drought (DT), waterlogging (WL), and exogenous ABA, respectively. The CD treatment involved putting the seedlings in a refrigerator at 0 °C for 1 hour. The DT seedlings were cultivated in soil with a 39% moisture content for 22 days. However, the WL seedlings were planted under the condition of soil moisture content of 100% for one hour. The ABA treatment involved spraying seedlings with exogenous ABA (150mg/L) for 3 days. Additionally, the control group seedlings were cultured at 24 °C, with a 61% soil moisture content. All treatments had three replications. Leaves of the treated seedlings were collected to investigate their expression patterns in response to the respective abiotic stresses. All samples were frozen in liquid nitrogen and stored at -80 °C for total RNA extraction.

### 2.4 Expression profile and co-expression analyses based on RNA-seq

Total RNA was extracted using the RNAprep Pure Plant Kit (Tiangen, Beijing, China). High-throughput sequencing was performed using the MGI platform, with PE150 reads length, by Annoroad Gene Technology Co., Ltd (Beijing, China). All clean reads were mapped to the N11-1 Chinese chestnut genome using the TopHat v2.1.1 software (Kim et al., 2013), and the number of reads mapped to each gene was counted using the HTSeq v0.11.3 software (Anders et al., 2015). The values of fragments per kilobase of the exon model per million mapped fragments (FPKM) were obtained through a perl script. Then, the differentially expressed genes (DEGs) were counted using DESeq2 with |log_2_(fold change)| ≥ 1 and FDR < 0.05 (Anders and Huber, 2010). The gene ontology (GO) enrichment analysis of DEGs was performed using TBtools software. The weighted correlation network analysis (WGCNA) was performed with all expressed genes (FPKM > 1) using the R package (Langfelder and Horvath, 2008), and the co-expression networks were generated using Cytoscape (Otasek et al., 2019).

### 2.5 Pollen magnetofection and drought treatment to positive transgenic Chinese chestnut seedlings

The full-length coding sequence (CDS) of the *CmPP2C31* with its termination codon was amplified from the *C. mollissima* cultivar ‘Yanbao’, and cloned into plasmid pBWA(V)HS to produce the *35S::CmPP2C31* vector. *Agrobacterium tumefaciens* (EHA105) with *35S::CmPP2C31* vector was transformed into ‘Yanbao’ Chinese chestnut seeds following a previously published protocol (Zhao et al., 2017). The magnetic nanoparticles (MNP) and plasmids (DNA) were mixed in a 1:1 mass ratio to obtain the MNP-DNA complex. Then, the MNP-NDA complex was mixed with Chinese chestnut pollen and incubated under a magnetic field. Finally, the pollen was given to the female flowers of ‘Yanbao’ to obtain transgenic Chinese chestnut seeds. The harvested Chinese chestnut seeds were cultivated into seedlings on the WPM medium containing 1.0 mg/L trans-Zeatin and 0.1 mg/L IAA at 25 °C for 30 days. After RT-qPCR verification, positive transgenic seedlings were propagated on a medium containing 1.0 mg/L trans-Zeatin, 0.2 mg/L brassinosteroid, 0.01 mg/L IAA, and 1.0 mg/L 6-benzylamino purine. Finally, PEG (15%) was added to the subculture medium to simulate drought, and the control was grown on a culture medium with no additions. Two weeks later, the contents of H_2_O_2_ (hydrogen peroxide) and MDA (malondialdehyde) were assayed following a previously described method (Sun et al., 2018).

### 2.6 Subcellular localization and yeast one hybridization

The CDS of *CmPP2C31* was cloned into plasmid 1300-GFP for generating the GFP-fused protein in living cells. *A. tumefaciens* (GV3101) containing the 1300-GFP-CmPP2C31 vector was used to inject the lower epidermis of tobacco (*Nicotiana benthamiana*) leaves. Subcellular localization of *CmPP2C31* was investigated at 72 h after infiltration. The fluorescence signal was detected using a laser copolymerization cross-fluorescence microscope at 488nm and 405nm excitation intensities for GFP and CFP, respectively.

The cDNA of *EVM0007407* was cloned by reverse transcription PCR of ‘Yanbao’ leaves. The cDNA was fused into the pGADT7 vector to construct the pGADT7-EVM0007407 recombinant plasmid. The *CmPP2C31* promoter (-2000bp) was cloned from ‘Yanbao’. Then, this promoter fragment was inserted into the pAbAi vector to construct the pCmPP2C31-AbAi recombinant plasmid. Y1H Gold yeast cells were co-transformed with pGADT7-EVM0007407 and pCmPP2C31-AbAi plasmids, and clones containing recombinant plasmids from SD/–Leu/–Ura medium were selected and grown in SD/–Leu/–Ura media containing different Aureobasidin A (AbA) concentrations (0ng/ml, 400ng/ml, 500ng/ml) to detected the interaction between EVM0007407 and *CmPP2C31-pro* (Yang et al., 2019).

## 3 Results

### 3.1 Identification and comprehensive characterization of *CmPP2C*s

We identified 68 genes from the whole genome of N11-1, a seedling Chinese chestnut cultivar (Wang J. et al., 2020). The 68 genes encode putative PP2C family proteins named *CmPP2C01* - *CmPP2C68* based on their locus on the chromosomes (Chr) (**Supplementary Figure S1**; **Supplementary Table S1**). The putative PP2C proteins ranged from 186 to 1079 amino acids (aa) length, with 20.31 kDa to 119.40 kDa predicted molecular weights. The theoretical isoelectric point (pI) of the CmPP2C proteins was 4.66 to 9.30 (**Supplementary Table S1**). Gene ontology (GO) analysis revealed that these *CmPP2C*s enriched ‘protein dephosphorylation’, ‘response to abscisic acid’, ‘response to water deprivation’, and ‘response to oxygen-containing compound’ (**Supplementary Figure S2**; **Supplementary Table S2**). Therefore, CmPP2Cs may regulate stress resistance in Chinese chestnut by regulating protein phosphorylation modifications.

**Figure 1:**
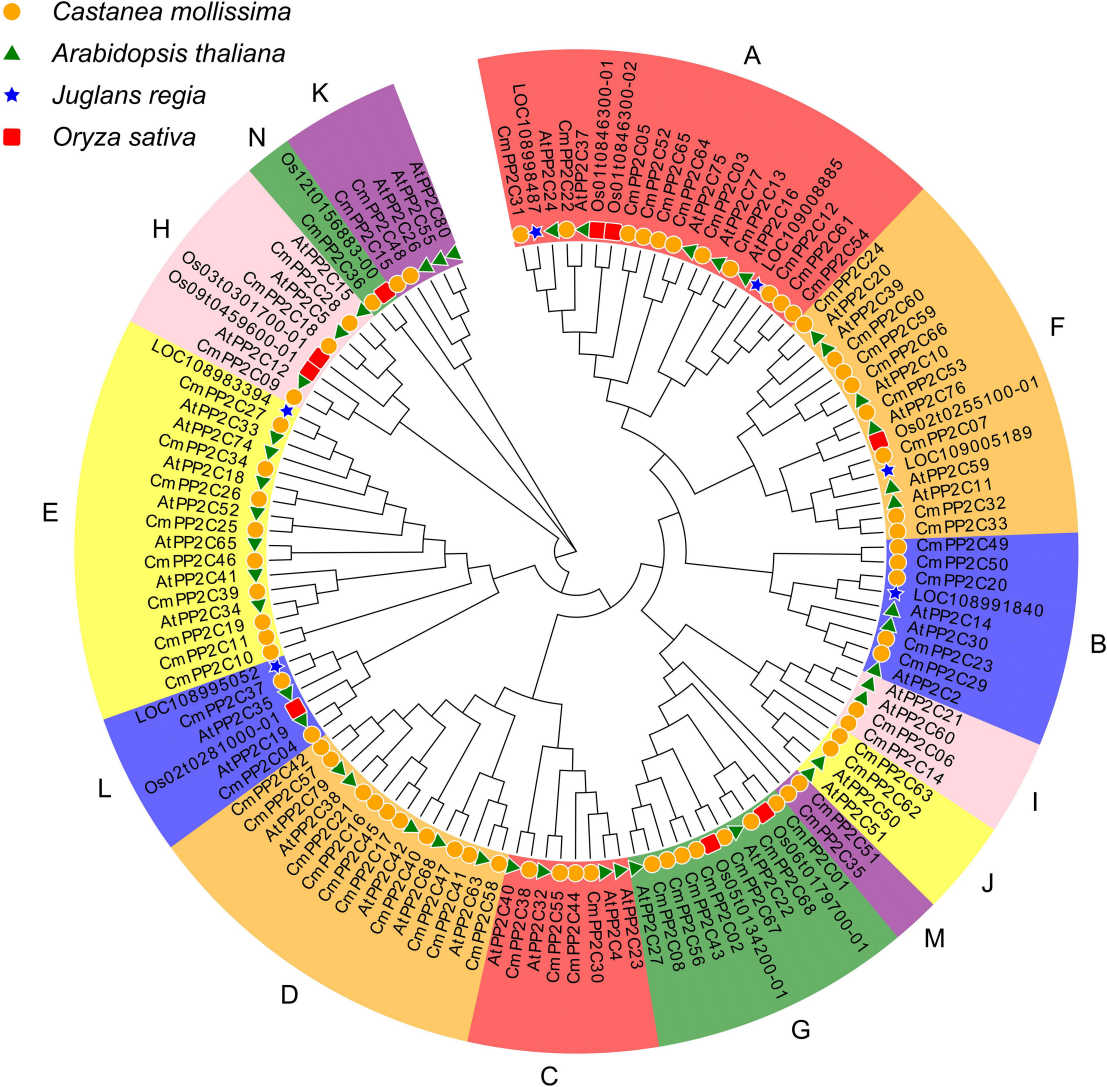
Neighbor-joining phylogenetic tree of PP2C proteins in *Castanea mollissima* (Chinese chestnut), *Arabidopsis thaliana*, *Juglans regiam* (walnut), and *Oryza sativa* (rice). The capital letters **A–N** represent the 14 subgroups of CmPP2C proteins.

To investigate the classification and evolutionary relationships of CmPP2C proteins, we constructed an unrooted phylogenetic tree based on the alignments of full-length protein sequences from Chinese chestnut, *Arabidopsis*, walnut, and rice. The CmPP2Cs were divided into 14 subgroups (A–N) according to their orthologs in *Arabidopsis* (**Figure 1**) (Xue et al., 2008). The distribution of PP2C from rice and walnut in different subgroups indicated that different types of PP2C maintained similar functions in species evolution. There were the most (11) CmPP2Cs in subgroup A (**Figure 1**; **Supplementary Table S4**). These members should have similar functions and might have evolved with the expansion of gene families caused by genome replication events. However, there was only one member in the N subgroup, CmPP2C36, which may have an independent evolutionary trajectory from other members. Further, the ten conserved motifs of CmPP2C proteins were identified, and showed clade-specificity. Motifs 1, 2, 3, 5, and 8 were widely distributed among CmPP2C proteins and formed two combinations (1-8-3-2 and 5-8-3-2), the possible core domains of the PP2C family (**Supplementary Figures S3**, **S4**; **Supplementary Tables S3**, **S4**). Similarly, *CmPP2C* genes from same subgroup had similar exon-intron structure and differ between subgroups (**Supplementary Figure S3C**; **Supplementary Table S4**). These indicated that the evolution and divergence of CmPP2Cs might have occurred at an early stage.

### 3.2 Analyses of expansion dynamics in Chinese chestnut and synteny between genomes of *PP2C*s based on comparative genomics

**Figure 2:**
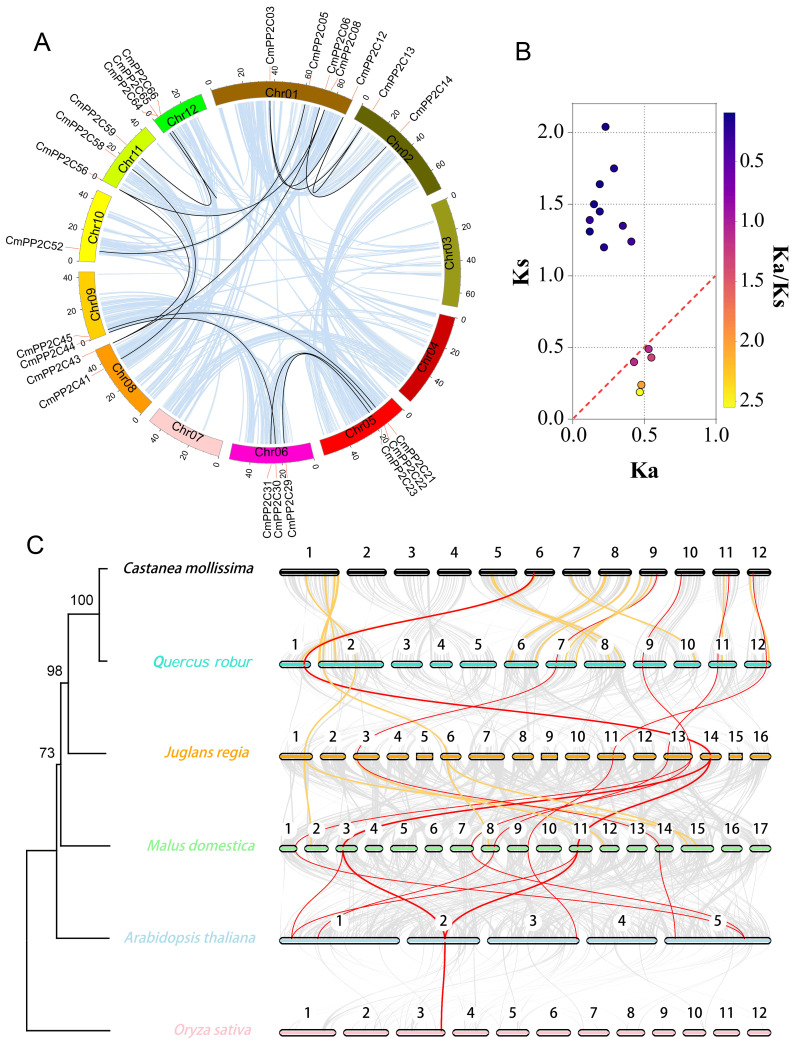
Collinearity analysis of Chinese chestnut *PP2C* family genes. **(A)** Lightblue lines indicate all synteny blocks in the Chinese chestnut genome, and black lines indicate duplicated *PP2C* gene pairs. **(B)** The scatter plot shows the Ka and Ks values of 15 *PP2C* gene pairs. The red dashed line indicates equal Ka and Ks values. **(C)** Synteny of *PP2C* gene pairs among six plant species. Red and orange lines indicate synteny genes; red lines represent collinear gene pairs among ≥ 5 genomes. Grey lines indicate collinear blocks between two adjacent genomes. The colored bars represent chromosomes of different species. The left tree illustrates the phylogenetic relationships between the six species.

Whole-genome duplication (WGD) analysis revealed synteny relationships among 15 pairs of *CmPP2Cs* genes across nine chromosomes (**Figure 2A**). In particular, *CmPP2C64* and *CmPP2C65* located in 8,039 bp apart of Chr12, indicating a likely tandem duplication event (**Supplementary Figure S1**; **Table 1**). These observations suggested that both segmental and tandem duplication possibly expanded the *CmPP2C* gene family. Furthermore, the values of Ka, Ks, and Ka/Ks ratios were calculated to estimate the dates of duplication events of *CmPP2C* gene pairs. The divergence time varied from 6.18 to 67.86 million years ago (MYa), spanning the Neogene (2.6-23.3 MYa) and Paleogene (23.3-68.5 MYa) periods. Five pairs of duplicated *CmPP2C*s might have been positively selected at the most recent divergence (6.18-16.24 MYa (Ka/Ks > 1)). Conversely, ten pairs might have undergone purifying selection in the early stages (40.14-67.86 MYa). The Ka/Ks ratios of *CmPP2C59/66* and *CmPP2C06/14* were both 0.09, indicating that they underwent the highest selection pressure to adapt to the natural environment in Paleogene (**Figure 2B**; **Table 1**). Therefore, we could speculate that natural environmental pressure (or abiotic stress) promoted the duplication of the *CmPP2C*s.

**Table 1:** Estimated duplication dates in the PP2C gene family of chestnut.

Duplicated Gene Pairs		Subgroup	Ka	Ks	Ka/Ks	Selection Type	Time (MYa)	Duplicated Type
gene1	gene2							
*CmPP2C22*	*CmPP2C31*	A	0.23	2.04	0.12	Purify	67.86	Segmental
*CmPP2C05*	*CmPP2C52*	A	0.29	1.75	0.17	Purify	58.31	Segmental
*CmPP2C21*	*CmPP2C45*	D	0.19	1.64	0.12	Purify	54.73	Segmental
*CmPP2C58*	*CmPP2C41*	D	0.15	1.50	0.10	Purify	49.96	Segmental
*CmPP2C08*	*CmPP2C56*	G	0.19	1.45	0.13	Purify	48.34	Segmental
*CmPP2C59*	*CmPP2C66*	F	0.12	1.39	0.09	Purify	46.26	Segmental
*CmPP2C03*	*CmPP2C12*	A	0.35	1.35	0.26	Purify	45.01	Segmental
*CmPP2C06*	*CmPP2C14*	I	0.12	1.31	0.09	Purify	43.63	Segmental
*CmPP2C64*	*CmPP2C65*	A	0.41	1.24	0.33	Purify	41.38	Tandem
*CmPP2C08*	*CmPP2C43*	G	0.22	1.20	0.18	Purify	40.14	Segmental
*CmPP2C03*	*CmPP2C13*	A	0.53	0.49	1.09	Positive	16.24	Segmental
*CmPP2C30*	*CmPP2C44*	C	0.55	0.43	1.30	Positive	14.20	Segmental
*CmPP2C12*	*CmPP2C13*	A	0.43	0.40	1.09	Positive	13.31	Segmental
*CmPP2C23*	*CmPP2C29*	B	0.48	0.24	2.01	Positive	8.02	Segmental
*CmPP2C56*	*CmPP2C43*	G	0.47	0.19	2.55	Positive	6.18	Segmental

Five comparative syntenic maps of Chinese chestnut association with *Quercus robur* (oak), *Juglans regia* (walnut), *Malus domestica* (apple), *Arabidopsis thaliana*, and *Oryza sativa* (rice) were constructed to reveal the evolutionary relationships of the *PP2C* gene family among different species (**Figure 2C**; **Supplementary Figure S5**). We found that 51 *CmPP2Cs* with corresponding orthologous genes in the other five genomes. Forty *CmPP2C*s (especially *CmPP2C31*) were syntenic in at least two genomes, indicating their crucial role in the *PP2C* family evolution (**Supplementary Table S5**). Furthermore, 22 *CmPP2C*s showed syntenic relationships with oak *PP2C* genes, 30 with walnut, 44 with apple, and 17 with *Arabidopsis*. Chinese chestnut and rice (the only monocot) shared eight *CmPP2C* gene pairs, which was much less than between Chinese chestnut and the four dicots. These results may suggest that most orthologous pairs occurred after the divergence of dicotyledons and monocotyledons. The phylogenetic tree of the *PP2C* family orthologous genes (**Figure 2C**; **Supplementary Table S6**) supported this inference.

### 3.3 Expression patterns of *CmPP2C*s and co-expression networks related to four stress treatments

**Figure 3:**
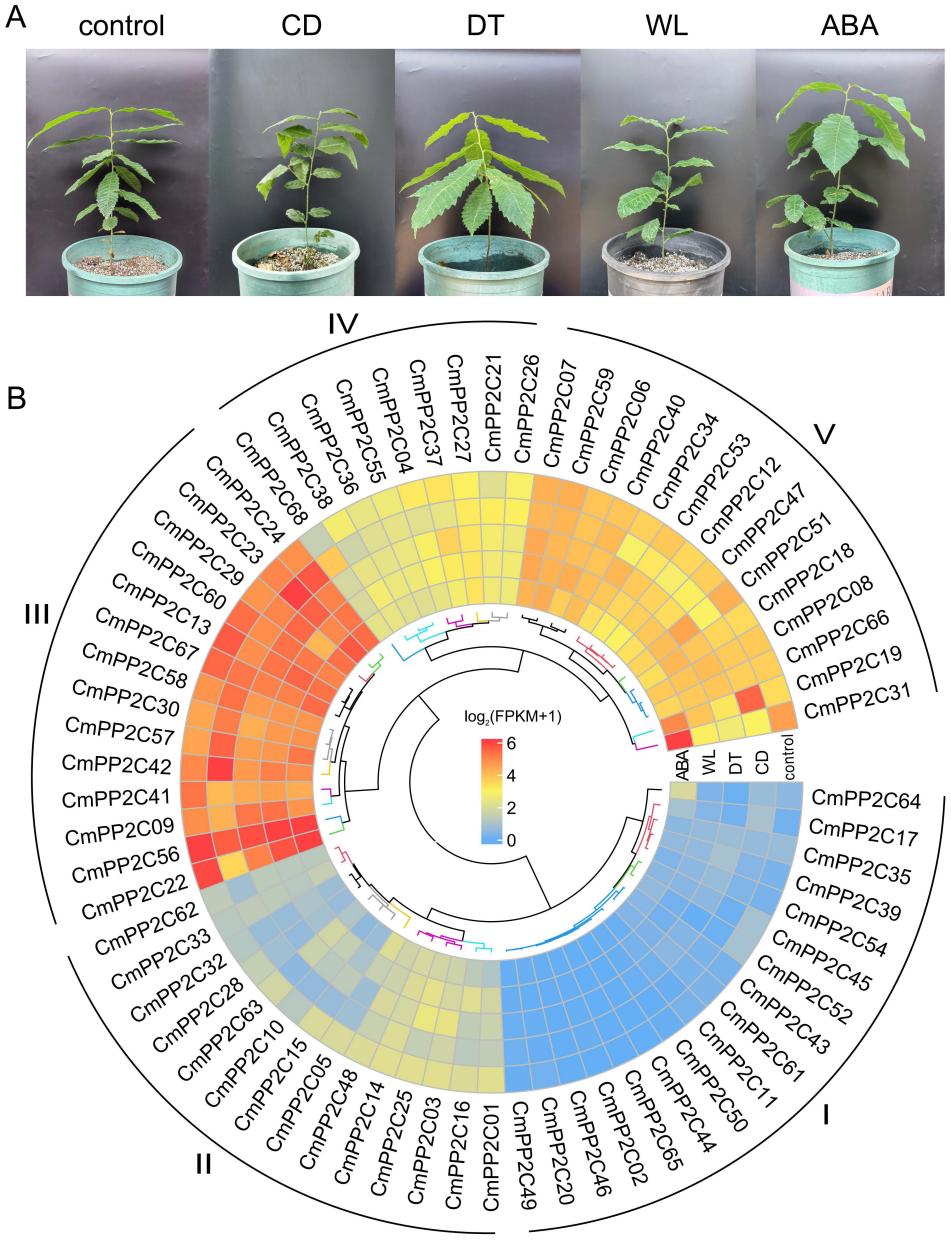
One-year-old ‘Yanbao’ seedlings were used for RNA-seq **(A)** and *CmPP2C* expression under different treatments **(B)**. CD, cold (low temperature); DT, drought; WL, waterlogging; ABA, exogenous ABA. The heatmap shows the expression of *CmPP2C* genes under different treatments. The colors correspond to the log_2_(FPKM+1) values. I, II, III, IV, and V represent *CmPP2C* subclades based on expression levels.

The transcriptome of chestnut seedling leaves from CD, DT, WL, ABA, and control treatments (with three replications) revealed the roles of *CmPP2C*s in stress resistance and signal transduction (**Figure 3A**; **Supplementary Figure S6**). RNA-seq generated 5.16-7.07 gigabases (Gb) clean data from each of the 15 libraries (**Supplementary Table S7**). The Chinese chestnut N11-1 genome contained 33,597 annotated genes, and 14,116 genes were almost non-expressed (FPKM < 0.1) across the 15 samples, which included eight *CmPP2C*s (**Supplementary Table S8**). High Pearson correlation coefficients (r > 0.74) indicated high-quality control among the biological replicates (**Supplementary Table S9**). The hierarchical clustering tree divided *CmPP2C* genes into five subclades based on their expression profiles. Subclade I had the greatest number (17) of *CmPP2C*s, but the *CmPP2C*s had the lowest expression (FPKM < 1) across treatments. In contrast, the 14 members of subclade III had the highest FPKM values (10.80 ≤ FPKM ≤ 86.80). The *CmPP2C*s in subclade V also showed relatively high expression levels (5.41 ≤ FPKM ≤ 79.63). Interestingly, *CmPP2C19* and *CmPP2C31* expression were the most sensitive to different treatments (Fold change > 3 or < 0.33) in subclade V (**Figure 3B**; **Supplementary Tables S8**, **S10**). Additionally, the relative expression levels measured by RT-qPCR confirmed the FPKM patterns of five *CmPP2C*s, with high correlation relationship (r > 0.86) (**Supplementary Figure S7**).

Furthermore, a total of 6,240 DEGs were identified in the CD, DT, WL, and ABA treatments by comparing with the control (**Supplementary Figure S8**; **Supplementary Table S8**). Twenty-two *CmPP2C*s were included in DEGs. Seven *CmPP2C*s were significantly up-regulated, and 14 were significantly down-regulated in one or more treatments. Interestingly, ABA significantly up-regulated *CmPP2C31*, and CD and DT significantly down-regulated it (**Figure 3B**; **Supplementary Tables S8, S10**).

**Figure 4:**
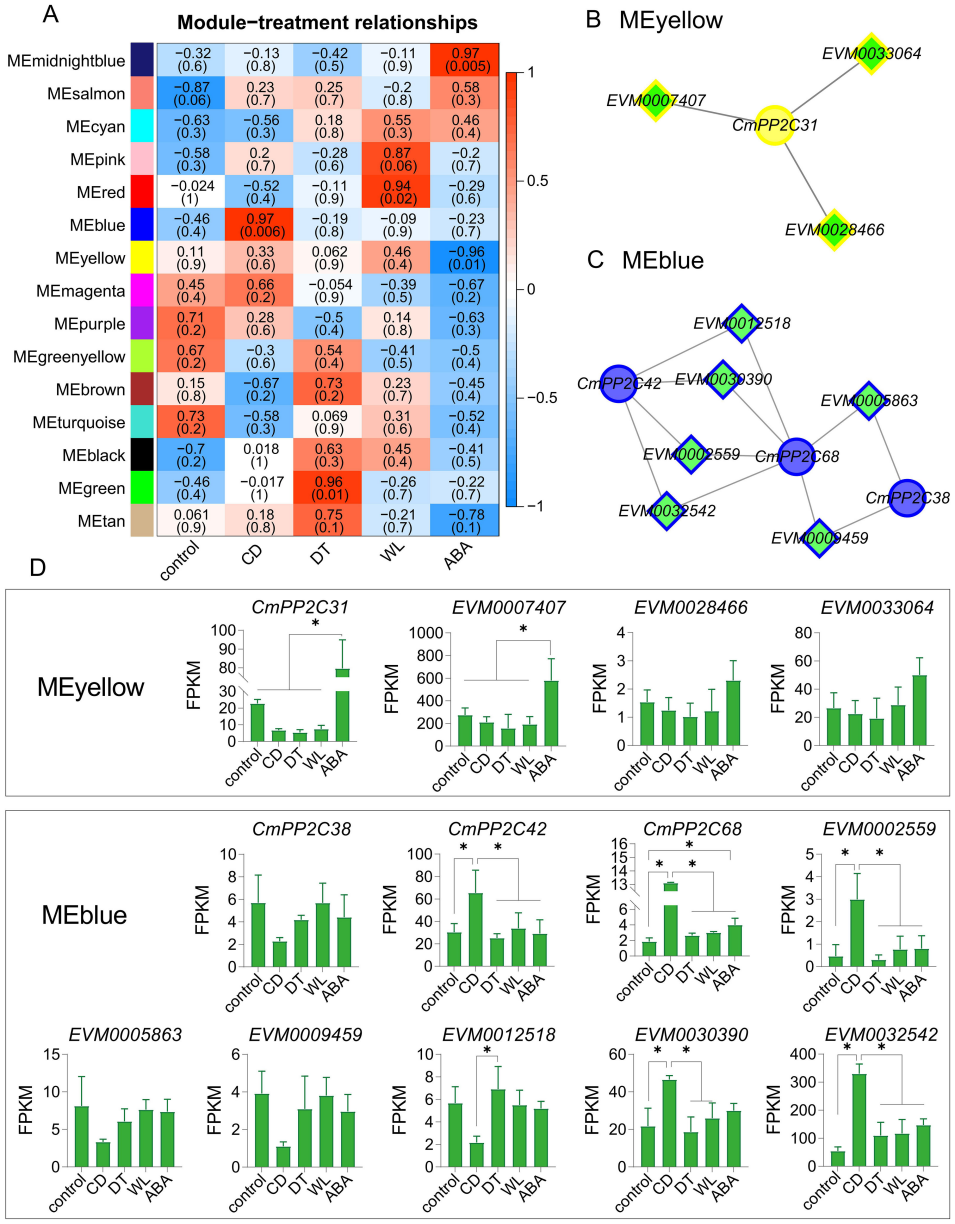
WGCNA module-trait relationships, co-expression networks, and module-specific gene expression profiles. **(A)** The heatmap represents relationships between WNCGA modules and treatments. The top and bottom (shown in parentheses) numerals in each cell represent the correlation coefficients and p-values, respectively. The meanings represented by CD, DT, WL, and ABA are the same as in **Figure 3**. **(B, C)** The networks represent co-expression relationships of *CmPP2C*s and transcription factors (TFs) in the MEyellow **(B)** and MEblue **(C)**. Diamonds represent TFs; circles indicate *CmPP2C*s. **(D)** The column diagrams describe the expression profiles of genes in panels **(B, C)**. *indicates significant differences at *P* < 0.05, as determined using Student’s t-test.

The WGCNA analysis using the 14,184 genes (FPKM > 1) identified 15 co-expression modules, with gene numbers ranging from 49 (MEmidnightblue) to 3,346 (MEturquoise) (**Supplementary Figure S9**; **Supplementary Table S8**). Five key modules (MEblue, MEgreen, MEred, MEmidnightblue, and MEyellow) were significantly associated with stress (|r| ≥ 0.94 and p < 0.05). MEmidnightblue was positively related to exogenous ABA treatment (r = 0.97), and MEyellow showed the opposite results (r = -0.96). MEblue, MEgreen, and MEred modules were positively related to CD (r = 0.97), DT (r = 0.96), and WL stresses (r = 0.94), respectively (**Figure 4A**). Furthermore, the module eigengene-based connectivity (KME, |KME| > 0.85) and the weight value (> 0.25) between pairwise genes revealed hub *CmPP2C*s in five key modules. MEblue had three hub *PP2Cs* (*CmPP2C38*, *CmPP2C42*, and *CmPP2C68*) and MEyellow had one (*CmPP2C31*). MEred, MEgreen, and MEmidnightblue no hub *CmPP2C* gene. All selected *CmPP2C*s were differentially expressed (**Figure 4B**; **Supplementary Table S8**).

The promoters of *CmPP2C31*, *CmPP2C38*, *CmPP2C42*, and *CmPP2C68* contained at least one stress-related *cis*-element, including ACE, ABRE, G-box, MYC, and MYB (**Supplementary Table S11**). Further, MEyellow and MEblue contained three (*EVM0007407*, *EVM0033064* and *EVM0028466*) and six (*EVM0002559*, *EVM0005863*, *EVM0009459*, *EVM0012518*, *EVM0030390* and *EVM0032542*) transcription factors (TF), respectively. These TFs could bind to the *cis*-acting elements and were significantly co-expressed with *CmPP2C31*, *CmPP2C38*, *CmPP2C42*, and *CmPP2C68* (**Figure 4B**; **Supplementary Figures S10**, **S11**). Especially, *EVM0007407* (*NAC072* gene) and *EVM0032542* (*NAC083* gene) showed the highest expression in MEyellow and MEblue and were co-expressed with *CmPP2C31* and *CmPP2C42*/*68*, respectively (**Figure 4C**; **Supplementary Figure S7**). These findings suggested that the hub *CmPP2C*s in the key modules, regulated by their upstream TFs, may participate in the stress response pathways of Chinese chestnut seedlings.

### 3.4 Validation of *CmPP2C31* enhancing drought resistance in chestnuts through pollen magnetofection

**Figure 5:**
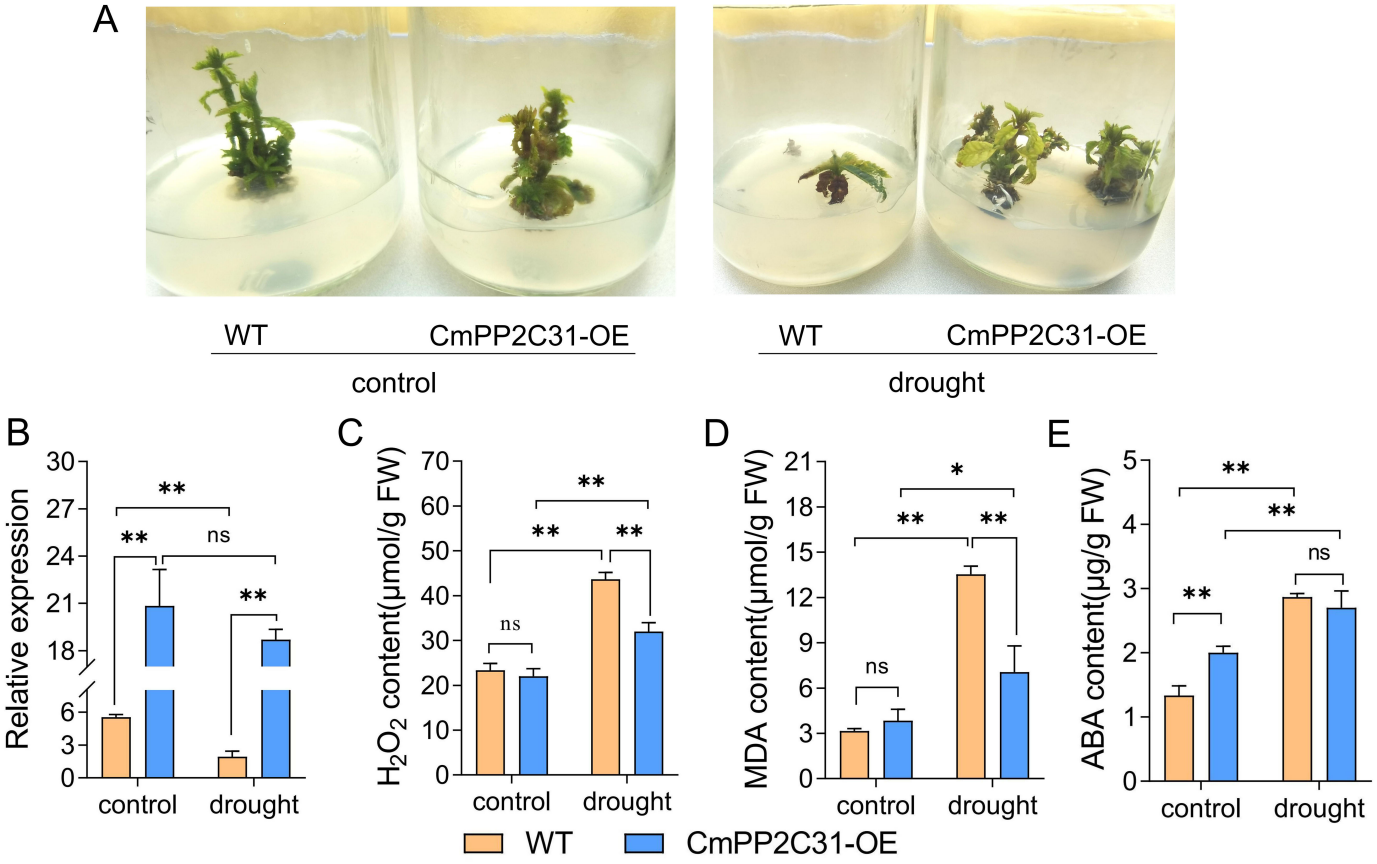
*CmPP2C31* regulates drought resistance in chestnut. **(A)** The growth of wild-type and over-expressing *CmPP2C31* Chinese chestnut seedlings under normal conditions and drought stress. WT: wild type line; CmPP2C31-OE: over-expressing *CmPP2C31* line. **(B)** The relative expression of *CmPP2C31* in WT and CmPP2C31-OE chestnuts under normal and drought stress conditions. **(C–E)** The contents of H_2_O_2_ **(C)**, MDA **(D)**, and endogenous ABA **(E)** in tissue culture seedling. * and ** indicate significant differences at *P* < 0.05 and *P* < 0.01, respectively, as determined using Student’s t-test.

*CmPP2C31* was the only up-regulated *PP2C* in ABA, the only down-regulated in CD/DT treatments, and the only hub *CmPP2C* in MEyellow. Thus, transgenic experiment by pollen magnetofection (Zhao et al., 2017) were conducted to explore its function in drought stress resistance. The over-expression vector *35S::CmPP2C31* was generated and then transformed into the seed of ‘Yanbao’ Chinese chestnut. We cultured the embryos of transgenic seeds into seedlings under tissue culture conditions, and 15% PEG was added to the subculture medium to simulate drought stress (**Figure 5A**). The relative expression of *CmPP2C31* was significantly increased in CmPP2C31-OE than the WT under normal and drought stress conditions (**Figure 5B**). Drought stress significantly inhibited the growth of Chinese chestnut seedlings and significantly increased the H_2_O_2_ and MDA content. However, over-expressing *CmPP2C31* in Chinese chestnut seedlings significantly reduced these trends (**Figure 5**). Furthermore, we found that over-expression of *CmPP2C31* was able to increase endogenous ABA content through feedback regulation (**Figure 5E**). These results indicated that *CmPP2C31* improved the tolerance of Chinese chestnut to drought stress by affecting ABA signaling transduction.

### 3.5 Subcellular localization of CmPP2C31 and the interaction with TF EVM0007407

**Figure 6:**
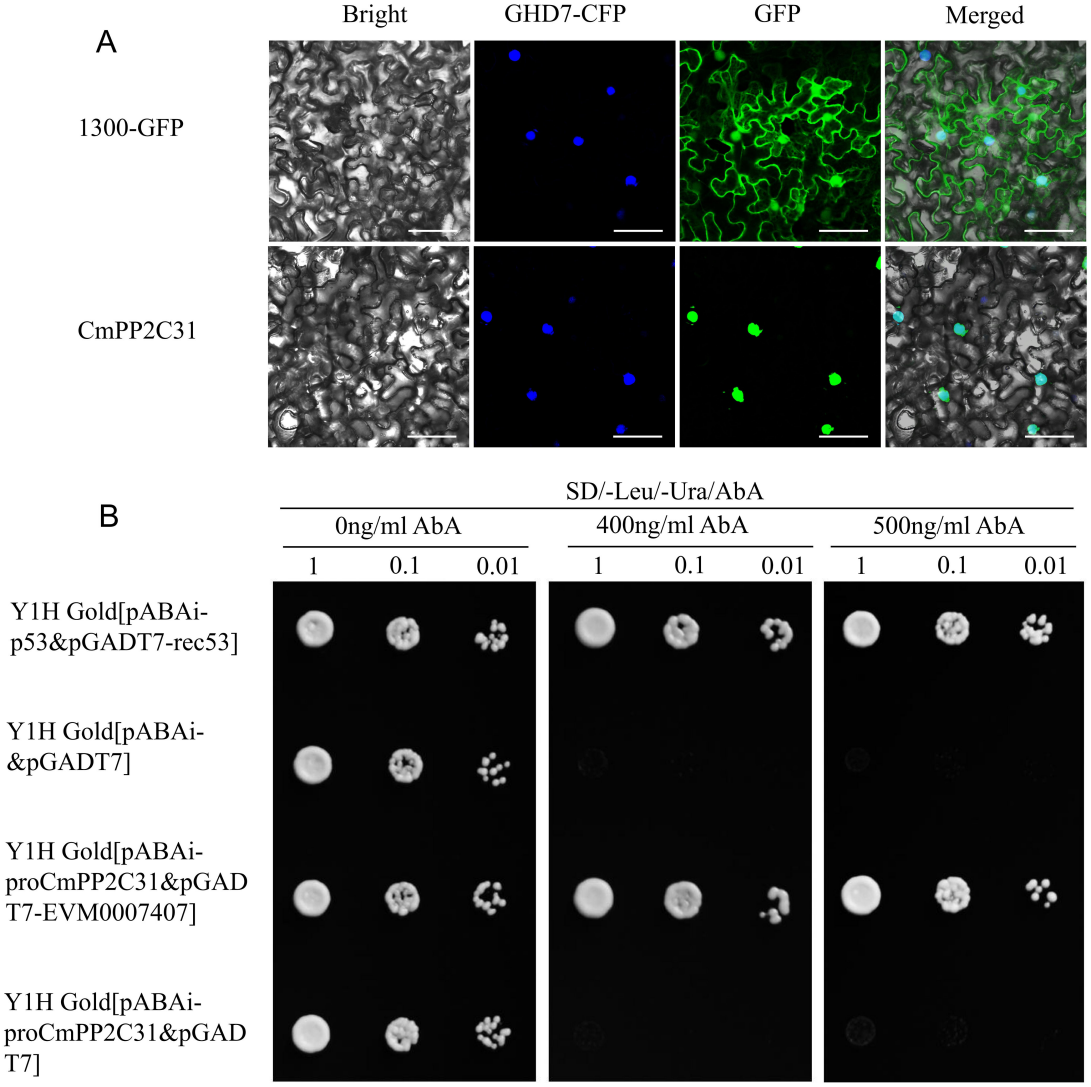
Subcellular localization of CmPP2C31 and the interactions between *CmPP2C31* and EVM0007407. **(A)** Subcellular localization of CmPP2C31. GHD7-CFP: cyan fluorescence of nuclear marker GHD7 fused with cyan fluorescent protein. GFP: green fluorescence of 1300-GFP and 1300-GFP-CmPP2C31. Bar=25 μm. **(B)** Detection of interactions between *CmPP2C31* and EVM0007407. Y1H revealed that EVM0007407 interacts with the CmPP2C31 promoter. The yeast strain of pABAi-proCmPP2C31&pGADT7-EVM0007407 is the experimental group. The yeast strain of pABAi-p53&pGADT7-rec53 is a positive control. The yeast strain of pABAi-&pGADT7 or pABAi-proCmPP2C31&pGADT7 is the negative control. All yeast strains were selected on SD/–Leu/–Ura media at different Aureobasidin A (AbA) concentrations (0, 400, and 500 ng/ml). The numbers (1, 0.1, and 0.01) on the top represent the dilution ratios of the yeast solution.

Subcellular PP2C localization and interaction with TF EVM0007407 revealed its regulation mechanism in drought resistance. The full-length CDS of *CmPP2C31* was fused to the C-terminus of green fluorescent protein (GFP). The GFP-fusion proteins and GFP alone were transiently expressed in *N. benthamiana*. The nucleus was identified by fusing the nuclear marker protein GHD7 with the cyan fluorescent protein (CFP). The fluorescent signals of GFP-fusion proteins were exclusively restricted to the nucleus, fully overlapping with the fluorescence of GHD7-CFP (**Figure 6A**), indicating that CmPP2C31 is localized in the nucleus. In contrast, the GFP signal alone was in both the nucleus and cytosol.

The protein of EVM0007407 is highly homologous to AtNAC072 of *Arabidopsis* and is the TF most likely regulating *CmPP2C31* expression under abiotic stress (**Figures 4B, D**). A Y1H identified the potential interaction between EVM0007407 and the promoter of *CmPP2C31*. Yeast strains containing pGADT7-EVM0007407&pABAi-ProPP2C31 grew normally in media containing 400 and 500 ng/ml Aureobasidin A (AbA) (**Figure 6B**). Therefore, EVM0007407 could directly bind to the promoter of *CmPP2C31*.

## 4 Discussion

The *PP2C* genes encode crucial signaling molecules in developmental processes, phytohormone signaling, and stress responses in plants (Kerk et al., 2002; Wei and Pan, 2014; Wang et al., 2021). At present, they have been identified and functionally studied in multiple species, such as *Arabidopsis*, rice (Xue et al., 2008), soybean (Shen et al., 2022), tomato (Qiu et al., 2022), potato (Wang Y. et al., 2020), apple, Chinese white pear (Wang et al., 2021), walnut (Chen et al., 2022), and jute (Pan et al., 2024). However, the Chinese chestnut *PP2C* gene family had not been characterized despite the availability of the complete genome assembly. Thus, this study identified 68 *CmPP2C*s at genome-wide, and comprehensively analyzed the *CmPP2C* gene family.

Normally, gene family expansion is driven by tandem and segmental duplication events (Wu et al., 2019; Duan et al., 2022; Zhang et al., 2023). In this study, collinearity analysis identified 14 segmental duplicate *CmPP2C* gene pairs and one tandem gene pair (**Table 1**). Therefore, segmental duplication is the initial driving force that expanded the *CmPP2C* family in chestnut. Among these duplicated gene pairs, those undergoing purification and positive selection displayed a clear time boundary. Ten *CmPP2C* gene pairs underwent strong purifying selection during the Paleogene period (40.14 - 67.86 Mya). In the Neogene period following the divergence of *C. mollissima* and *Q. robur* (~18.3 Mya) (Wang J. et al., 2020), five *CmPP2C* pairs genes in the Chinese chestnut genome underwent positive selection to promote adaptation to new environmental pressures (**Figure 2B**; **Table 1**). Syntenic analysis between species showed that orthologous *CmPP2C*s in dicotyledonous plants were significantly higher than those in monocotyledonous plants. Walnuts and apples had more orthologues than *Arabidopsis*, probably because the walnut genome experienced WGD events (Zhang et al., 2020), while the apple genome originated from heteroploidy events (Velasco et al., 2010).

Phylogenetic analysis categorized Chinese chestnut PP2C proteins into 14 subgroups (A - N) following the classification in *Arabidopsis* (Xue et al., 2008). The clustered PP2Cs have highly similar amino acid sequences, indicating they play similar functions. Further, clade specific conserved motifs and gene structures between different subgroups can cause functional differentiation of proteins (Han et al., 2018; Jia et al., 2018; Zheng et al., 2020). Previous studies have shown that AtPP2C16, AtPP2C37, and AtPP2C77 encoded by *HYPERSENSITIVE TO ABA1* (*HAB1*), *AtPP2CA*, and *ABSCISIC ACID-INSENSITIVE 2* (*ABI2*), respectively, are important for blocking the ABA signaling pathway in protoplast (Yoshida et al., 2006; Xue et al., 2008). Therefore, the CmPP2C proteins in subgroup A, especially CmPP2C03, CmPP2C13, CmPP2C22, and CmPP2C31, may also negatively regulate ABA signal transduction (**Figure 1**). The CmPP2Cs in subgroups B and F possibly regulate salt stress, as AtPP2C20 (AtPPC3, an isozyme of phosphoenolpyruvate carboxylase) and AtPP2C30 (AtHPP2C5) are reportedly involved in this process (Xue et al., 2008; Connell et al., 2018; Zang et al., 2019). Similarly, CmPP2C30/38/44 in subgroup C can regulate meristem or leaf development, as AtPP2C4 (PLL4), AtPP2C23 (PLL5), and AtPP2C32 (POL) possess these functions (Song and Clark, 2005; Xue et al., 2008).

Hormones like ABA and MeJA may regulate *PP2Cs*, and these genes are closely related to various stresses, such as drought, salicylic acid, low-temperature, anaerobic induction, and defense responsiveness (Khan et al., 2019; Wang et al., 2021; Chen et al., 2022; Wu et al., 2022). *CmPP2Cs* might participate in these bio-processes. The *CmPP2C*s in subgroup A contained the most stress-related *cis*-acting elements (46), including 12 ABRE (ABA), 11 ARE (anaerobic induction), five MBS (drought), four CCAAT-boxes (drought), six TCA-elements (salicylic acid), five LTR-elements (low-temperature), and three TC-rich repeats (defense) (**Supplementary Table S11**). Furthermore, the transcriptome of CD, DT, WL, ABA, and CK Chinese chestnut seedlings revealed DEGs and GO terms relating to defense response, protein phosphorylation, and response to hormones like ABA. The role of ABA signals in stress-responsive mechanisms is widely reported, especially for the *PP2Cs* in subgroup A. At least six *AtPP2Cs* in *Arabidopsis* are homologous to subgroup A *CmPP2C*s, increasing ABA sensitivity under various stresses (Merlot et al., 2001; Umezawa et al., 2009). Similarly, the *PbrPP2C*s in pears showed substantial transcriptional variations in response to ABA treatment under drought, NaCl, heat, and cold stresses (Wang et al., 2021). The *ZmPP2C-A10* in maize was a negative regulation factor of ABA stress response (Xiang et al., 2017).

Previous studies have demonstrated that ABA is a key signaling factor in plant responses to abiotic stresses, and the *PP2Cs* genes participate in abiotic stress response through an ABA-dependent pathway (Hu et al., 2017; Tischer et al., 2017; Chen et al., 2022). In this study, WGCNA analysis revealed five key modules, including four hub *CmPP2C*s, that are significantly associated with stress. *CmPP2C31*, the only up-regulated *PP2C* in ABA and down-regulated in CD/DT treatments, participated in the response, especially to cold and drought stress. In general, abiotic stress can increase the ABA content in plants, activating or inhibiting the expression of related genes. This leads the regulation of downstream gene expression consistent between abiotic stress and exogenous ABA treatments. But, the expression of *CmPP2C31* was not the case. We speculate that this may be due to the inhibition of the expression of *CmPP2C31* by MYB (encoded by *EVM0017160*, *EVM0027174*, *EVM0032234*, *EVM0031270*, or *EVM0017310*) and its recruited HDA (histone deacetylase: encoded by *EVM0000576*, *EVM0022926*, or *EVM0030963*), which are significantly upregulated under stress conditions (**Supplementary Figures S12**, **S13**) (Nguyen et al., 2019). *CBF* (C-repeat binding factor: encoded by *EVM0010161* or *EVM0022102*) may also be a factor that inhibits the expression of *CmPP2C31* under stress conditions (Cui et al., 2013). The detailed reasons still need further research. Moreover, over-expressing *CmPP2C31* enhanced the drought resistance of Chinese chestnut seedlings (**Figure 5**), similar to the role of *ZmPP2C15* (Pang et al., 2024). This regulatory mechanism might follow an ABA-dependent pathway, which was similar to the findings in other plants (Hu et al., 2017; Tischer et al., 2017; Chen et al., 2022). The findings of this study indicate the general function of the Chinese chestnut *PP2C* gene family. However, the individual gene functions at the molecular level remain unknown.

The PP2C protein mainly localizes in the nucleus, which modulates protein kinase signaling and regulates various stress responses by interacting with TFs (Valdés et al., 2012; Nguyen et al., 2019). In this study, the subcellular localization experiment confirmed that CmPP2C31 performed functions in the nucleus (**Figure 6A**). The WGCNA analysis showed that the TF gene of *EVM0007407* was the highest expressed in MEyellow and co-expressed with *CmPP2C31* (**Figure 4C**). The Y1H assay confirmed that the TF EVM0007407 directly binds to the *CmPP2C31* promoter, which had been predicted by the *cis*-acting element analysis (**Figure 6B**; **Supplementary Figure S9**). The *NAC31* gene in *Picea wilsonii* (homologous to *AtNAC072*) cooperates with the ABA-dependent pathway gene, *DREB2A*, and modulates drought resistance in transgenic *Arabidopsis* (Huang et al., 2024). *EVM0007407* was highly homologous to *AtNAC072* (Hickman et al., 2013). The ABA-dependent pathway may also achieve the interaction between *EVM0007407* and *CmPP2C31* in regulating drought and cold resistance in chestnut. This study revealed the importance of the Chinese chestnut *CmPP2C* genes in abiotic stress responses, especially *CmPP2C31*, laying a foundation for further studies on the regulatory network of *CmPP2C* genes in abiotic stresses. Furthermore, it plays a vital role in mining excellent gene resources and improving the abiotic stress tolerance of crops.

## 5 Conclusion

This study identified 68 members of *PP2C* family in the Chinese chestnut genome. Segmental and tandem duplication both drove the expansion of this family to adapt to natural environmental pressures. *CmPP2C31, CmPP2C38*, *CmPP2C42*, and *CmPP2C68* were highly correlated with responses to abiotic stresses in Chinese chestnut seedlings. This study also demonstrated that CmPP2C31 is a nuclear protein, and TF EVM0007407 regulates *CmPP2C31* expression by binding to its promoter. Besides, over-expressing *CmPP2C31* could significantly enhance drought resistance in Chinese chestnut seedlings. These findings indicate that *CmPP2C* genes especially *CmPP2C31* play potential vital roles in chestnut response to abiotic stresses and lay a foundation for further molecular characterization of resistance to abiotic stress in Chinese chestnut.

## Data Availability

The clean sequence data of transcriptome reported in this paper have been deposited in the Genome Sequence Archive (Chen et al., 2021) in National Genomics Data Center (CNCB-NGDC Members and Partners, 2022), China National Center for Bioinformation/Beijing Institute of Genomics, Chinese Academy of Sciences (GSA: CRA017593) that are publicly accessible at https://ngdc.cncb.ac.cn/gsa. The reference genome of the N11-1 Chinese chestnut (Wang J. et al., 2020) used in this study were obtained from the Genome Warehouse in BIG Data Center under accession number GWHANWH00000000 (https://bigd.big.ac.cn/gwh). The sequences of primers and plasmid vectors used in this study are listed in **Supplementary File S1**.
