# Supplementary material for: Identification and characterization of CmPP2C31 playing a positive role in the abiotic stress resistance of Chinese chestnut via an integrated strategy
Source: Front Plant Sci. 2024 Dec 13;15:1491269. doi: 10.3389/fpls.2024.1491269 (PMC11671270; doi:10.3389/fpls.2024.1491269)
Supplement: Supplementary file 1 [file DataSheet1.pdf]

Supplementary file 1:

The primers used for cloning the CDS sequence of *CmPP2C31*:
CmPP2C31_F: ATGGCGGAGATCTGCTGTGGATT
CmPP2C31_R: CTACGTGTCCCTTCTTAGGTCTAC

The primers used for cloning the promoter sequence of *CmPP2C31*:
CmPP2C31-pro_F: TTCATTTTCTGGAGGAGC
CmPP2C31-pro_R: TCCACCGTTGATCTATGC

The primers used for cloning the CDS sequence of transcription factor *EVM0007407*:
EVM0007407_F: ATGGGAGTACCGGAGACAGAC
EVM0007407_R: TCACGGCCTAAATCCGAACC

CmPP2C31 over-expression vector used for pollen magnetolysis transgenic method:

> pBWA(V)HS-OE-CmPP2C31-GLosgfp

tagaatagcatcggtaacatgagcaaagtctgccgccttacaacggctctcccgctgacgccgtcccggactgatgggctgcctgtatcgagtggtg
attttgtgccgagctgccggtcggggagctgttggctggctggtggcaggatatattgtggtgtaaacaaattgacgcttagacaacttaataacacat
tgcggacgtttttaatgttagactgaattaacgccgaattaattcgggggatctggattttagtactggattttggttttaggaattagaaattttattgatag
aagtattttacaaatacaaatacatactaagggtttcttatatgctcaacacatgagcgaaaccctataggaaccctaattcccttatctgggaactactc
acacattattatggagaaactcgagcttgtcgatcgacagatccggtcggcatctactctatttctttgccctcggacgagtgctggggcgtcggtttcc
actatcggcgagtacttctacacagccatcggtccagacggccgcgcttctgcgggcgatttgtgtacgcccgacagtcccggctccggatcggac
gattgcgtcgcatcgaccctgcgcccaagctgcatcatcgaaattgccgtcaaccaagctctgatagagttggtcaagaccaatgcggagcatatac
gcccggagtcgtggcgatcctgcaagctccggatgcctccgctcgaagtagcgcgtctgctgctccatacaagccaaccacggcctccagaagaa
gatgttggcgacctcgtattgggaatccccgaacatcgcctcgctccagtcaatgaccgctgttatgcggccattgtccgtcaggacattgttggagc
cgaaatccgcgtgcacgaggtgccggacttcggggcagtcctcggcccaaagcatcagctcatcgagagcctgcgcgacggacgcactgacgg
tgtcgtccatcacagtttgccagtgatacacatggggatcagcaatcgcgcatatgaaatcacgccatgtagtgtattgaccgattccttgcggtccga
atgggccgaacccgctcgtctggctaagatcggccgcagcgatcgcatccatagcctccgcgaccggttgtagaacagcgggcagttcggtttca
ggcaggtcttgcaacgtgacaccctgtgcacggcgggagatgcaataggtcaggctctcgctaaactccccaatgtcaagcacttccggaatcgg
gagcgcggccgatgcaaagtgccgataaacataacgatctttgtagaaaccatcggcgcagctatttacccgcaggacatatccacgccctcctac
atcgaagctgaaagcacgagattcttcgccctccgagagctgcatcaggtcggagacactgtcgaacttttcgatcagaaacttctcgacagacgtc
gcggtgagttcaggctttttcatatctcattgccccccggatctgcgaaagctcgagagagatagatttgtagagagagactggtgatttcagcgtgt
cctctccaaatgaaatgaacttccttatatagaggaaggtcttgcgaaggatagtgggattgtgcgtcatcccttacgtcagtggagatatcacatcaat
ccacttgctttgaagacgtggttggaacgtcttctttttccacgatgctcctcgtgggtgggggtccatctttgggaccactgtcggcagaggcatcttg
aacgatagcctttcctttatcgcaatgatggcatttgtaggtgccaccttccttttctactgtccttttgatgaagtgacagatagctgggcaatggaatcc
gaggaggtttcccgatattaccctttgttgaaaagtctcaatagccctttggtcttctgagactgtatctttgatattcttggagtagacgagagtgtcgtg
ctccaccatgttatcacatcaatccacttgctttgaagacgtggttggaacgtcttctttttccacgatgctcctcgtgggtgggggtccatctttgggacc
actgtcggcagaggcatcttgaacgatagcctttcctttatcgcaatgatggcatttgtaggtgccaccttccttttctactgtccttttgatgaagtgaca
gatagctgggcaatggaatccgaggaggtttcccgatattaccctttgttgaaaagtctcaatagccctttggtcttctgagactgtatctttgatattcttg
gagtagacgagagtgtcgtgctccaccatgttggcaagctgctctagccaatacgcaaaccgcctgcaggtctagacatggagtcaaagattcaaat
agaggacctaacagaactcgccgtaaagactggcgaacagttcatacagagtctcttacgactcaatgacaagaagaaaatcttcgtcaacatggtg
gagcacgacacacttgtctactccaaaaatatcaaagatacagtctcagaagaccaaagggcaattgagacttttcaacaaagggtaatatccggaa
acctcctcggattccattgcccagctatctgtcactttattgtgaagatagtggaaaaggaaggtggctcctacaaatgccatcattgcgataaaggaa
aggccatcgttgaagatgcctctgccgacagtggtcccaaagatggacccccacccacgaggagcatcgtggaaaaagaagacgttccaaccac
gtcttcaaagcaagtggattgatgtgatatctccactgacgtaagggatgacgcacaatcccactatccttcgcaagacccttcctctatataaggaag
ttcatttcatttggagagaacacgggggactttgcaacATGGCGGAGATCTGCTGTGGATTGGTGAACGAGAGCG
AGACATCAACGGCGTGTGAGCAGAGCTCCCGAGCGGCAAGGCGGAGGAGAATGGAGATC
AGACGGTTCAAATTCGTAGCCGGCGTGGCCACACCGGAGCCAGAGAGTACTAGTACTGAG
CATAAGCGTCAGAAGCTAGAAGGCTATTACACGGCGTCGTTTTCTCGTGACTGCGAGTACG
CCGTAGAGACGTCCGTTACAGACGAGGAGAAGAAAGTAGTAGAAAACGAGAGCCTCGAG
ACCAAAGGTATCTCGAATTCGAATTCGAATGATGGTTGTGTTTCAACTTCGGTTTCGAGTG
AGTATCCGAAATTCGGTGTTGCATCTGTGTGTGGAAGACGGAGAGACATGGAAGACGCTG
TGGCTATACACCCTTCGTTCTCAAGCCATGATCACAATTCCACAACGAAATTGCATTACTTC
GGAGTTTACGACGGCCATGGCTGCTCTCACGTGGCAACGAAGTGTAGAGAAAAGTTACAT
GAGCTGGTGAAGGAAGAGTTGTTATCGTTAGAGACGCAAGAGGATGAGTGGAAAGGAGT
GATGGAGCGAAGCTTTTATCGCATGGACAAGGAAGTGAATGCATGGAACGAGACCGTTTT
GGGTGCAAAGTGTAGGTGTGAGCTTCAATCTCCAGAGTGTGATGCGGTTGGATCTACTGCG

GTTGTTGCAATTGTGACACCCAATAAGATCATCGTTGCCAATTGTGGTGATTCAAGAGCTG
TGCTTTGCCGAAACGGCAAGCCTGTACCTCTTTCCTCCGATCACAAGCCGGATCGTCCGGA
CGAGTTGAATCGGATCCAAGCCGCAGGTGGTCGGGTTATCTACTGGGACGGCCCACGTGT
CCTCGGAGTTCTCGCCATGTCTAGAGCTATTGGTGATAACTATCTGAAGCCTTATGTGAGCT
GTGAGCCGGAGGTGACGATAACGGATCGGACGGCGGAGGACGACTGCCTGATTCTGGCG
AGTGATGGGCTTTGGGACGTGGTGTCAAATGAGACGGCGTGTGGGGTGGCGCGTATGTGC
TTAAGAGGGAAAGCACACGCGCCTCCGTGTTCTCCGCCCGGTGCTGAGGTGGCTGTTTCC
GAAACATCAGACAAAGCGTGTTCGGACGCGTCGATGTTGCTGACGAAGTTGGCCTTGGCT
AGGCAAAGTCCTGACAACGTTAGCGTGGTCGTGGTAGACCTAAGAAGGGACACGtgtacagcg
gccgcttcagggagtggttccggaagcggctctggatcgggctcagggagtggttccggcagcggctctggatcggctgccgcagcaatttgtat
cgtgaagggcgaggaactcttcaccggcgtggtgccgatcctcgtggagctcgacggcgacgtgaacggccacaagttctccgtgtccggcgag
ggcgagggcgacgccacctacggcaagctcaccctcaagttcatctgcaccaccggcaagctcccggtgccgtggccgaccctcgtgaccacct
tctcctacggcgtgcagtgcttctcccgctacccggaccacatgaagcgccacgacttcttcaagtccgccatgccggagggctacgtgcaggagc
gcaccatcttcttcaaggacgacggcaactacaagacccgcgccgaggtgaagttcgagggcgacaccctcgtgaaccgcatcgagctcaaggg
catcgacttcaaggaggacggcaacatcctcggccacaagctcgagtacaactacaactcccacaacgtgtacatcatggccgacaagcagaaga
acggcatcaaggccaacttcaagacccgccacaacatcgaggacggcggcgtgcagctcgccgaccactaccagcagaacaccccgatcggc
gacggcccggtgctcctcccggacaaccactacctctccacccagagcgccctctccaaggacccgaacgagaagcgcgaccacatggtgctcc
tcgagttcgtgaccgccgccggcatcacccacggcatggacgagctctacaactgatgcaggtgagagcacctcacctgcaaaatgtaactagctc
tgtcttcagtactgggcccgaagactgaccagctcgaatttccccgatcgttcaaacatttggcaataaagtttcttaagattgaatcctgttgccggtctt
gcgatgattatcatataatttctgttgaattacgttaagcatgtaataattaacatgtaatgcatgacgttatttatgagatgggtttttatgattagagtcccg
caattatacatttaatacgcgatagaaaacaaaatatagcgcgcaaactaggataaattatcgcgcgcggtgtcatctatgttactagatcgggccatc
cgcactgtagcggatggcctaaaaaaaaaactagaagagacgagtctgagactcagcgtctcggtcgcagtcataacttcgtatagcatacattata
cgaagttatgggccgcattaccctgttatccctaggccgcataacttcgtatagcctacattataggatggagggatatcctctcttaaggtagcgagc
aagctctaagaggagtgtcgacaagcttggcactggccgtcgttttacaacgtcgtgactgggaaaaccctggcgttacccaacttaatcgccttgca
gcacatccccctttcgccagctggcgtaatagcgaagaggcccgcaccgatcgcccttcccaacagttgcgcagcctgaatggcgaatgctagag
cagcttgagcttggatcagattgtcgtttcccgccttcagtttaaactatcagtgtttgacaggatatattggcgggtaaacctaagagaaaagagcgttt
attagaataacggatatttaaaagggcgtgaaaaggtttatccgttcgtccatttgtatgtgcatgccaaccacagggttcccctcgggatcaaagtact
ttgatccaacccctccgctgctatagtgcagtcggcttctgacgttcagtgcaggagatgatcgcggccgggtacgtgttcgagccgcccgcgcatg
tctcaaccgtgcggctgcatgaaatcctggccggtttgtctgatgccaagctggcggcctggccggccagcttggccgctgaagaaaccgagcgc
cgccgtctaaaaaggtgatgtgtatttgagtaaaacagcttgcgtcatgcggtcgctgcgtatatgatgcgatgagtaaataaacaaatacgcaaggg
gaacgcatgaaggttatcgctgtacttaaccagaaaggcgggtcaggcaagacgaccatcgcaacccatctagcccgcgccctgcaactcgccg
gggccgatgttctgttagtcgattccgatccccagggcagtgcccgcgattgggcggccgtgcgggaagatcaaccgctaaccgttgtcggcatcg
accgcccgacgattgaccgcgacgtgaaggccatcggccggcgcgacttcgtagtgatcgacggagcgccccaggcggcggacttggctgtgt
ccgcgatcaaggcagccgacttcgtgctgattccggtgcagccaagcccttacgacatatgggccaccgccgacctggtggagctggttaagcag
cgcattgaggtcacggatggaaggctacaagcggcctttgtcgtgtcgcgggcgatcaaaggcacgcgcatcggcggtgaggttgccgaggcg
ctggccgggtacgagctgcccattcttgagtcccgtatcacgcagcgcgtgagctacccaggcactgccgccgccggcacaaccgttcttgaatca
gaacccgagggcgacgctgcccgcgaggtccaggcgctggccgctgaaattaaatcaaaactcatttgagttaatgaggtaaagagaaaatgagc
aaaagcacaaacacgctaagtgccggccgtccgagcgcacgcagcagcaaggctgcaacgttggccagcctggcagacacgccagccatgaa
gcgggtcaactttcagttgccggcggaggatcacaccaagctgaagatgtacgcggtacgccaaggcaagaccattaccgagctgctatctgaata
catcgcgcagctaccagagtaaatgagcaaatgaataaatgagtagatgaattttagcggctaaaggaggcggcatggaaaatcaagaacaacca
ggcaccgacgccgtggaatgccccatgtgtggaggaacgggcggttggccaggcgtaagcggctgggttgtctgccggccctgcaatggcact
ggaacccccaagcccgaggaatcggcgtgacggtcgcaaaccatccggcccggtacaaatcggcgcggcgctgggtgatgacctggtggaga
agttgaaggccgcgcaggccgcccagcggcaacgcatcgaggcagaagcacgccccggtgaatcgtggcaagcggccgctgatcgaatccgc
aaagaatcccggcaaccgccggcagccggtgcgccgtcgattaggaagccgcccaagggcgacgagcaaccagatttttcgttccgatgctcta

tgacgtgggcacccgcgatagtcgcagcatcatggacgtggccgttttccgtctgtcgaagcgtgaccgacgagctggcgaggtgatccgctacg
agcttccagacgggcacgtagaggtttccgcagggccggccggcatggccagtgtgtgggattacgacctggtactgatggcggtttcccatctaa
ccgaatccatgaaccgataccgggaagggaagggagacaagcccggccgcgtgttccgtccacacgttgcggacgtactcaagttctgccggcg
agccgatggcggaaagcagaaagacgacctggtagaaacctgcattcggttaaacaccacgcacgttgccatgcagcgtacgaagaaggccaa
gaacggccgcctggtgacggtatccgagggtgaagccttgattagccgctacaagatcgtaaagagcgaaaccgggcggccggagtacatcga
gatcgagctagctgattggatgtaccgcgagatcacagaaggcaagaacccggacgtgctgacggttcaccccgattactttttgatcgatcccggc
atcggccgttttctctaccgcctggcacgccgcgccgcaggcaaggcagaagccagatggttgttcaagacgatctacgaacgcagtggcagcgc
cggagagttcaagaagttctgtttcaccgtgcgcaagctgatcgggtcaaatgacctgccggagtacgatttgaaggaggaggcggggcaggctg
gcccgatcctagtcatgcgctaccgcaacctgatcgagggcgaagcatccgccggttcctaatgtacggagcagatgctagggcaaattgccctag
caggggaaaaaggtcgaaaagatctctttcctgtggatagcacgtacattgggaacccaaagccgtacattgggaaccggaacccgtacattggga
acccaaagccgtacattgggaaccggtcacacatgtaagtgactgatataaaagagaaaaaaggcgatttttccgcctaaaactctttaaaacttatta
aaactcttaaaacccgcctggcctgtgcataactgtctggccagcgcacagccgaagctcccggatacggtcacagcttgtctgtaagcggatgcc
gggagcagacaagcccgtcagggcgcgtcagcgggtgttggcgggtgtcggggcgcagccatgacccagtcacgtagcgatagcggagtgta
tactggcttaactatgcggcatcagagcagattgtactgagagtgcaccatatgcggtgtgaaataccgcacagatgcgtaaggagaaaataccgc
atcaggcgttcatccgcttcctcgctcactgactcgctgcgctcggtcgttcggctgcggcgagcggtatcagctcactcaaaggcggtaatacggtt
atccacagaatcaggggataacgcaggaaagaacatgtgagcaaaaggccagcaaaaggccaggaaccgtaaaaaggccgcgttgctggcgtt
tttccataggctccgcccccctgacgagcatcacaaaaatcgacgctcaagtcagaggtggcgaaacccgacaggactataaagataccaggcgt
ttccccctggaagctccctcgtgcgctctcctgttccgaccctgccgcttaccggatacctgtccgcctttctcccttcgggaagcgtggcgctttctca
tagctcacgctgtaggtatctcagttcggtgtaggtcgttcgctccaagctgggctgtgtgcacgaaccccccgttcagcccgaccgctgcgccttat
ccggtaactatcgtcttgagtccaacccggtaagacacgacttatcgccactggcagcagccactggtaacaggattagcagagcgaggtatgtag
gcggtgctacagagttcttgaagtggtggcctaactacggctacactagaaggacagtatttggtatctgcgctctgctgaagccagttaccttcgga
aaaagagttggtagctcttgatccggcaaacaaaccaccgctggtagcggtggtttttttgtttgcaagcagcagattacgcgcagaaaaaaaggatc
tcaagaagatcctttgatcttttctacggggtctgacgctcagtggaacgaaaactcacgttaagggattttggtcatgcattctaggtactaaaacaatt
catccagtaaaatataatattttattttctcccaatcaggcttgatccccagtaagtcaaaaaatagctcgacatactgttcttccccgatatcctccctgat
cgaccggacgcagaaggcaatgtcataccacttgtccgccctgccgcttctcccaagatcaataaagccacttactttgccatctttcacaaagatgtt
gctgtctcccaggtcgccgtgggaaaagacaagttcctcttcgggcttttccgtctttaaaaaatcatacagctcgcgcggatctttaaatggagtgtcc
tcttcccagttttcgcaatccacatcggccagatcgttattcagtaagtaatccaattcggctaagcggctgtctaagctattcgtatagggacaatccga
tatgtcgatggagtgaaagagcctgatgcactccgcatacagctcgataatcttttcagggctttgttcatcttcatactcttccgagcaaaggacgcca
tcggcctcactcatgagcagattgctccagccatcatgccgttcaaagtgcaggacctttggaacaggcagctttccttccagccatagcatcatgtcc
ttttcccgttccacatcataggtggtccctttataccggctgtccgtcatttttaaatataggttttcattttctcccaccagcttatataccttagcaggagac
attccttccgtatcttttacgcagcggtatttttcgatcagttttttcaattccggtgatattctcattttagccatttattatttccttcctcttttctacagtatttaa
agataccccaagaagctaattataacaagacgaactccaattcactgttccttgcattctaaaaccttaaataccagaaaacagctttttcaaagttgtttt
caaagttggcgtataacatagtatcgacggagccgattttgaaaccgcggtgatcacaggcagcaacgctctgtcatcgttacaatcaacatgctacc
ctccgcgagatcatccgtgtttcaaacccggcagcttagttgccgttcttccgaatagcatcggtaacatgagcaaagtctgccgccttacaacggct
ctcccgctgacgccgt

CmPP2C31 over-expression vector for sub-cellular localization analysis:

>pCambia1300-35S-CmPP2C31-GFP

gaggtccaaagggcaattgggcttctcaacaacgggtaatttcgggaacctcctcggattccattgcccagttatctgtcactttattgcgacgatagtg
aaaaggaaggtggctcctacaaatgccatcattgcgataaaggaaaggccatcgttgaagatgcctctgccgacagtggtcccaaagatggaccct
cacccacgaggagcatcgtggaaaaagaagacgttccaaccacgtcttcaaagccaagtggattgatgtgatatctccactgacgtaagggatgac
gcacaatcccactatccttcgcaagacccttccttcaaaaaaaaggaagttcatttccatttggagagaacacgggggacgagctcggtaccATG
GCGGAGATCTGCTGTGGATTGGTGAACGAGAGCGAGACATCAACGGCGTGTGAGCAGAG
CTCCCGAGCGGCAAGGCGGAGGAGAATGGAGATCAGACGGTTCAAATTCGTAGCCGGCG
TGGCCACACCGGAGCCAGAGAGTACTAGTACTGAGCATAAGCGTCAGAAGCTAGAAGGCT
ATTACACGGCGTCGTTTTCTCGTGACTGCGAGTACGCCGTAGAGACGTCCGTTACAGACGA
GGAGAAGAAAGTAGTAGAAAACGAGAGCCTCGAGACCAAAGGTATCTCGAATTCGAATTC
GAATGATGGTTGTGTTTCAACTTCGGTTTCGAGTGAGTATCCGAAATTCGGTGTTGCATCTG
TGTGTGGAAGACGGAGAGACATGGAAGACGCTGTGGCTATACACCCTTCGTTCTCAAGCC
ATGATCACAATTCCACAACGAAATTGCATTACTTCGGAGTTTACGACGGCCATGGCTGCTC
TCACGTGGCAACGAAGTGTAGAGAAAAGTTACATGAGCTGGTGAAGGAAGAGTTGTTATC
GTTAGAGACGCAAGAGGATGAGTGGAAAGGAGTGATGGAGCGAAGCTTTTATCGCATGGA
CAAGGAAGTGAATGCATGGAACGAGACCGTTTTGGGTGCAAAGTGTAGGTGTGAGCTTCA
ATCTCCAGAGTGTGATGCGGTTGGATCTACTGCGGTTGTTGCAATTGTGACACCCAATAAG
ATCATCGTTGCCAATTGTGGTGATTCAAGAGCTGTGCTTTGCCGAAACGGCAAGCCTGTAC
CTCTTTCCTCCGATCACAAGCCGGATCGTCCGGACGAGTTGAATCGGATCCAAGCCGCAG
GTGGTCGGGTTATCTACTGGGACGGCCCACGTGTCCTCGGAGTTCTCGCCATGTCTAGAGC
TATTGGTGATAACTATCTGAAGCCTTATGTGAGCTGTGAGCCGGAGGTGACGATAACGGAT
CGGACGGCGGAGGACGACTGCCTGATTCTGGCGAGTGATGGGCTTTGGGACGTGGTGTCA
AATGAGACGGCGTGTGGGGTGGCGCGTATGTGCTTAAGAGGGAAAGCACACGCGCCTCCG
TGTTCTCCGCCCGGTGCTGAGGTGGCTGTTTCCGAAACATCAGACAAAGCGTGTTCGGAC
GCGTCGATGTTGCTGACGAAGTTGGCCTTGGCTAGGCAAAGTCCTGACAACGTTAGCGTG
GTCGTGGTAGACCTAAGAAGGGACACGgtcgacatggtgagcaagggcgaggagctgttcaccggggtggtgcccatc
cttcgagcgtgcccc

Vectors used for yeast one hybrid experiments:

>pAbAi-proPP2C31

gggcataggtctgtgctcttccttcgttcttccttctgttcggagattaccgaatcaaaaaaatttcaaggaaaccgaaatcaaaaaaaagaataaaaaa
aaaatgatgaattgaaaagcttCAATTTTTACAACCCTAGATAGATCCCACCGGACACGAGTCACTGCAT
CACTTACGTGTCCACCTCTTCCCGACACGTATCTCACATCACACTCCCTTTCCACACGTGTC
AAACATCCACACACACCTTGACCCAGCAGCACATGTTGTTGAGCTTGAGTTTGAGTTTGGT
AGGGTTTCAATTTCCCATTTCAAAATCGAAAGAATTAGTAATTTTTAACCTAACCTGACCTT
ACTAATACCACAGTACCCATACCATAAAAAGTAACCGTAGTTAGAGCCGTGAATGGAACTT
GTTGCCTGGTCACGTGGCAGGTGGCACTGACTTTGCCTCCTGTCAATTTCATGCAACAGAA
ATATATATATATAGTACAATTGCTCGCCAGCCAGATTTTCCACGTGGTACCCTTTCTAAGGCT
TGAGTGTTTTCATCCATGGCATGGCCCCCACAAGACGTACTGTCCTATCTGTCACTGCACA
CGTTCCCAATCCCCTCACACCACGTGTAAAAGAAATCTAGTTTATTGATTATGTTCCTGCCT
ACCTGAATCAAAACCTTTATCATTACTGACGTGTACGGTAGAGGTTATTTTAGACTTTTTAG
CCCAATTTAATATGGGGTCAATGAAAGGAGTCTAAGATTAAGACACGTGTCAAAAGATTAA
GAAAACCGCTTATAAATATTCCCATTAGGCGTCAACGATTGGGACACATGGGCCCACCTGT
TAAAATCGGAAACTGATACGTACAGGCACTTGATTGGACCACAAGAGCTGAGAGGAATAA
AGTTGGAATTTAAAAAAATTAAAAATTAAAACAACAATCGTGGCCGAGTCTAGTATCCCAC
GATGGAGGCACGTGTCAACAACTCTAAAATCTTTTTATTTAAAAAAAAAAAACAGAGAAA
AACCACGCCTAGTACGATTAGCGTAGGACCACATGCGTGTAAGGAAAGAAAGTTCATGTA
CTCAAAGGTGTTTAAATCCAATTGGACCACGTACCGCCGTACATGAAATTGAAGATACACG
TGGTTGATTTTAACGTAGGATAAAAGAGGAATACCGCATAGATCAACGGTGGAGATCTGTT
CTTTTAGGctcgaggcatgtgctctgtatgtatataaaactcttgtctctcttctctaaatctt

>pGADT7-EVM0007407

ccccgggggggaaccatacgacgtaccagattacgctcatatgATGGGAGTACCGGAGACAGACCCACTTTCTCAG
CTAAGCTTGCCACCTGGGTTTAGGTTCTTTCCGACTGATGAGGAGCTTTTGGTGCAATATCT
ATGTAGAAAAGTAGCCGGGCACCATTTCAATTTGCAAATTATTGGTGAAATTGATTTGTACA
AGTTTGATCCATGGGTGTTACCAAGCAAAGCTATATTTGGGGAAAAAGAATGGTATTTTTTT
AGTCCTAGAGACAGGAAGTACCCAAATGGATCGAGACCAAATAGGGTTGCTGGGTCTGGT
TATTGGAAGGCAACTGGGACTGACAAAGTTATCACCACCGAGGGTCGTAAAGTTGGTATTA
AGAAAGCTCTAGTATTCTACGTTGGAAAAGCCCCTAAAGGAACAAAAACTAATTGGATTAT
GCATGAGTATCGCTTATTAGAGCACTCACGAAAGAGTGGAAGCACTAAGTTGGATGATTGG
GTTTTATGTCGGATTTATAAGAAGAACTCGAGCGCTCAGAAGCCAATGACAAGTGTTTCAA
CTTCAAGCAAAGAACAAAGCAATGATTCATCGTCTTCATCTCACCTGGACGACATGTTGGA
CTCGTCGTTTCCTGAAATTAATGACAGGTTCTTCGCTTTGCCACGTGTGAATTCCCTCAAGA
ACTTTCAAAATGATGACAAGCTCAGTTTCCAGAATCTGGGTTCTGGGAATTTTGACTGGGC
CAGCCTTGCTGGGCTCAACTCGGTGCCTGAATTCGCTGTTGAAAACCAAGCTCATCAAGC
TCAGACACAGGGGATTGTGAATTACAACGGTAATGACGTTTATGTCCCTTCTATCCCACAG
CTTTGCCACGTGGATGAAGAGGTTCAGAGTGGACTCAGAACAAACTCGGGGATGTTTCAG
CAGAACTCGAACTCGAACTCGAACGTGTTGACACAGAACTACTCTAACTCGCCCGACCCA
TTTGGGTTTAGGTACCCAACTCAATCGGGTGGGTTCGGATTTAGGCCGTGActcgagctgcagatga
atcgtagatactaaaacccccgccagt
