# Supplementary material for: Identification and characterization of CmPP2C31 playing a positive role in the abiotic stress resistance of Chinese chestnut via an integrated strategy
Source: Front Plant Sci. 2024 Dec 13;15:1491269. doi: 10.3389/fpls.2024.1491269 (PMC11671270; doi:10.3389/fpls.2024.1491269)
Supplement: Supplementary file 2 [file Image1.pdf]

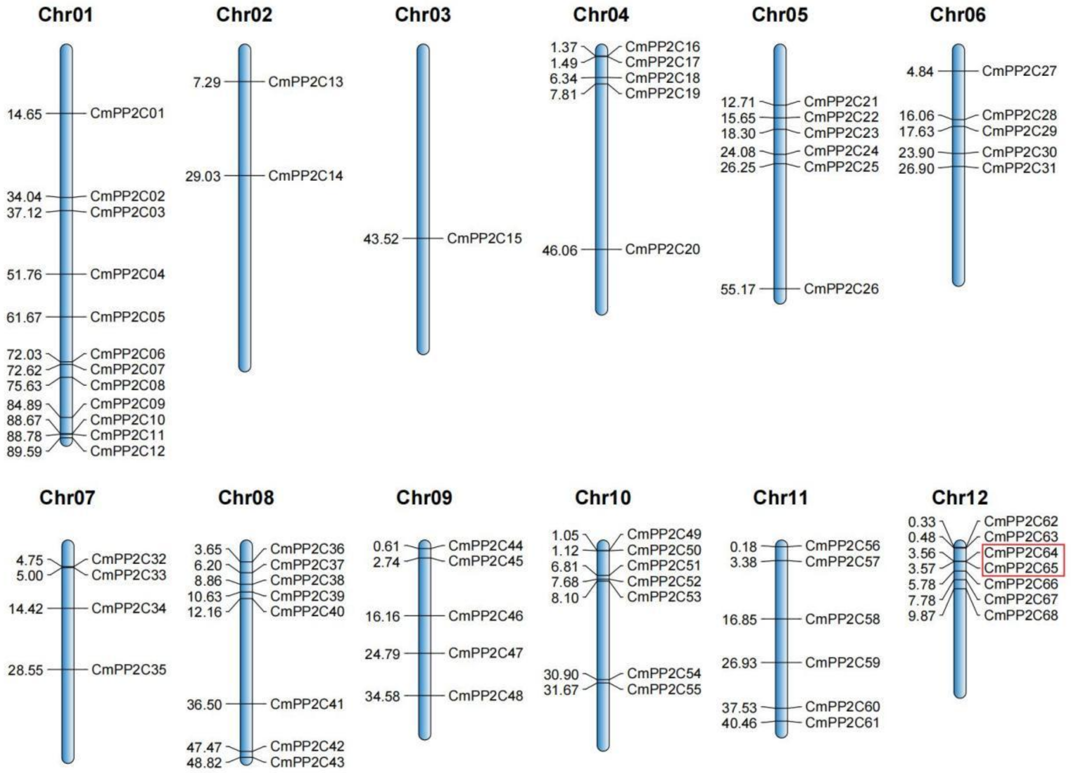

Figure S1. Chromosome distribution of Chinese chestnut PP2C family genes. The numbers on the left vertical bars indicate the physical positions (Mb) of *CmPP2Cs*, and corresponding gene names are marked on the right side. The two genes in the red rectangle have a tandem repeat relationship.

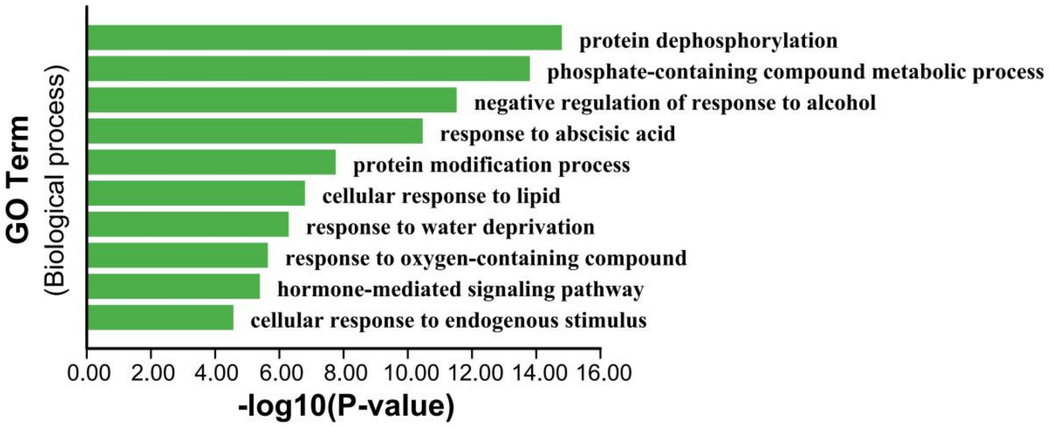

Figure S2. Representative terms in the GO enrichment results of CmPP2C protein.

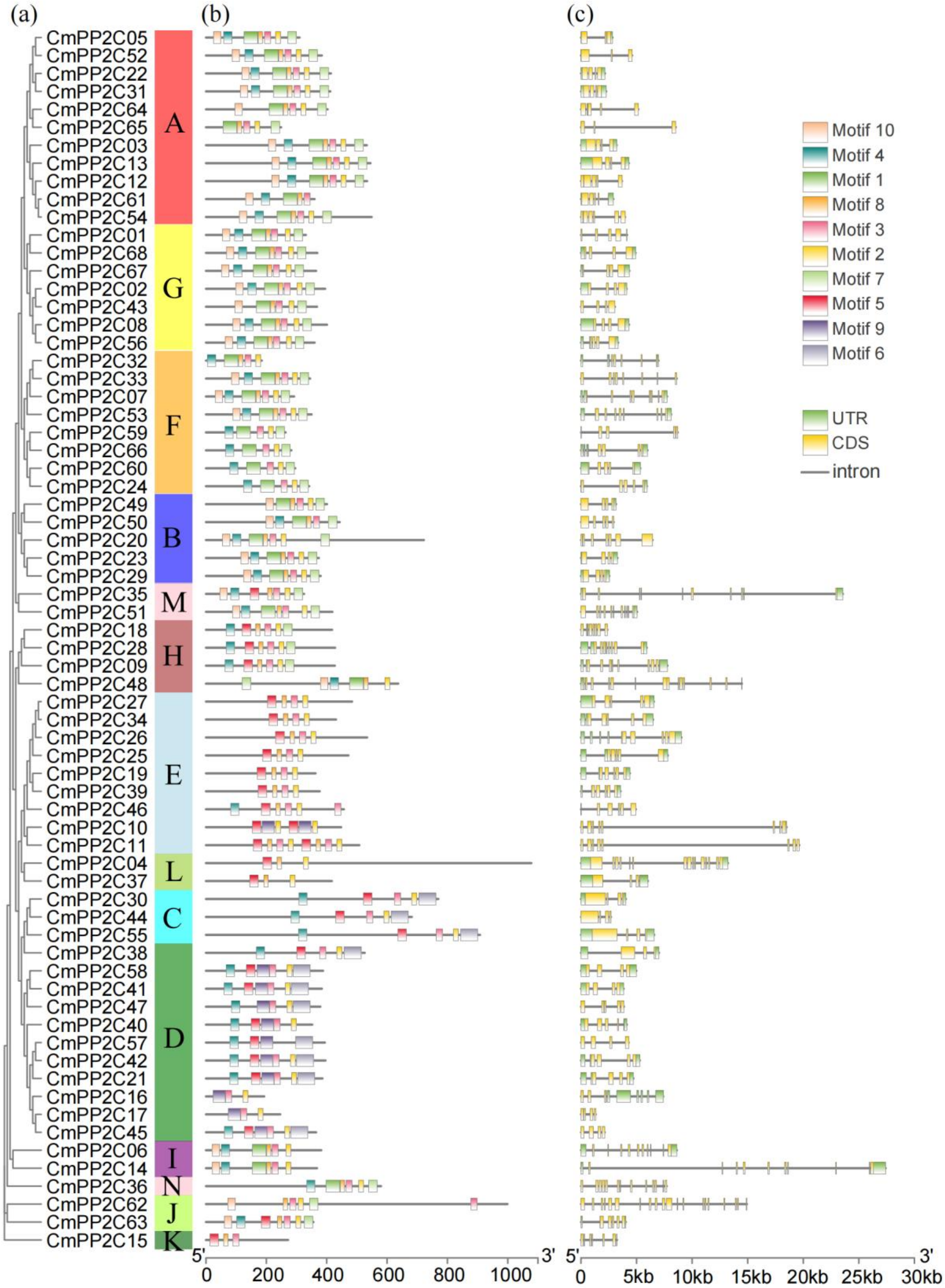

Figure S3. Conserved CmPP2C motif patterns and structure schematics. (a) A phylogenetic tree based on 68 CmPP2C protein sequences. (b) Conserved motifs of CmPP2C proteins with different colors depicting separate patterns. (c) Structure of *CmPP2C* genes.

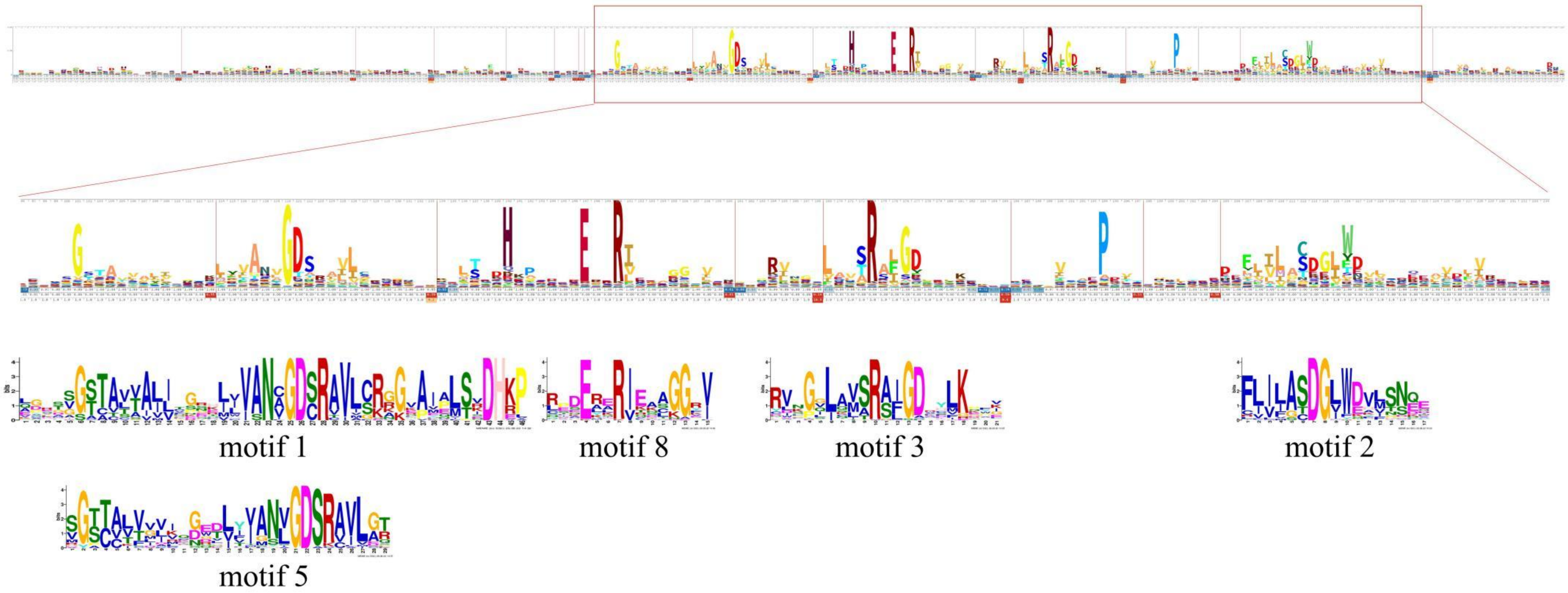

Figure S4. Correspondence between motifs and the core area of pp2c domain.

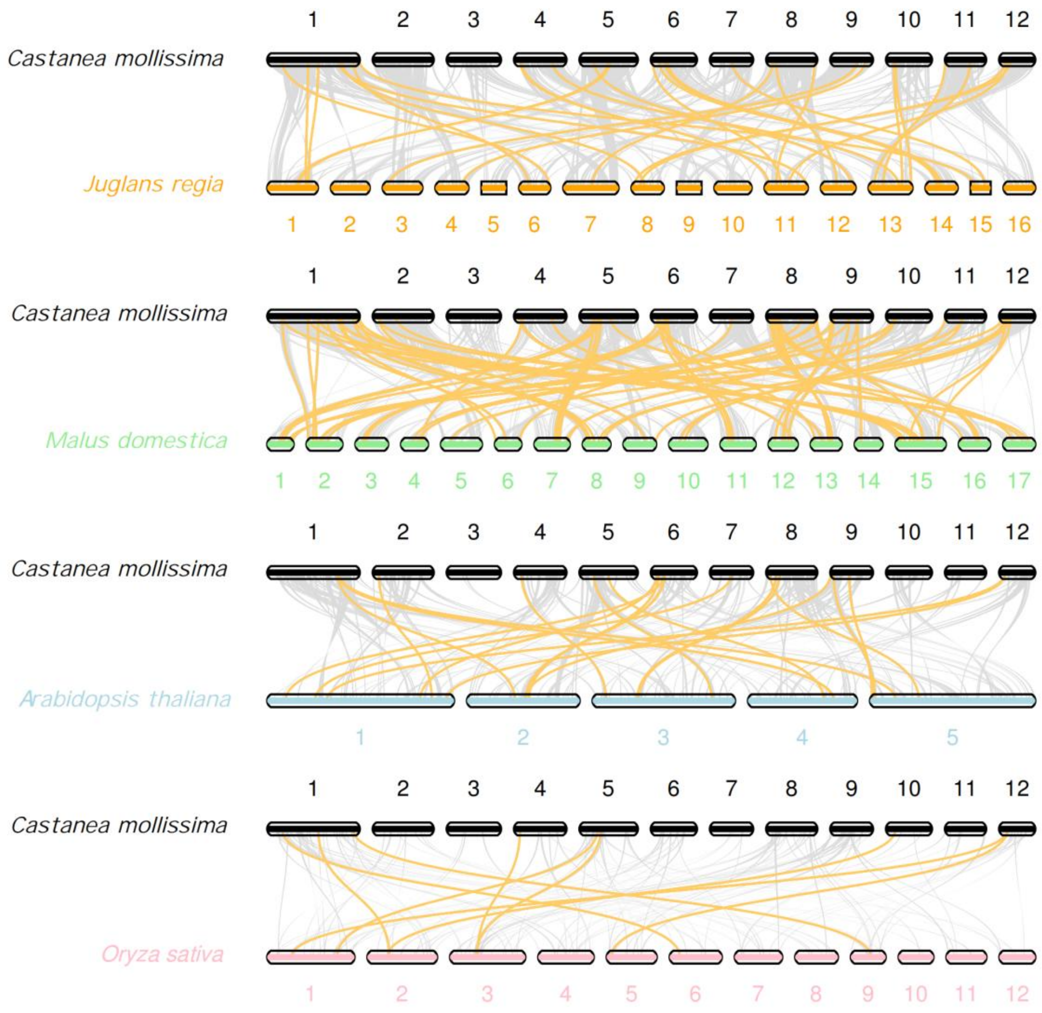

Figure S5. Synteny maps of the *PP2C* genes between chestnut and other four plant species. The grey lines in the background indicate the collinear blocks between the chestnut genome and other genomes. Bold, orange lines highlight the syntenic *PP2C* gene pairs. The colored bars represent chromosomes of different species.

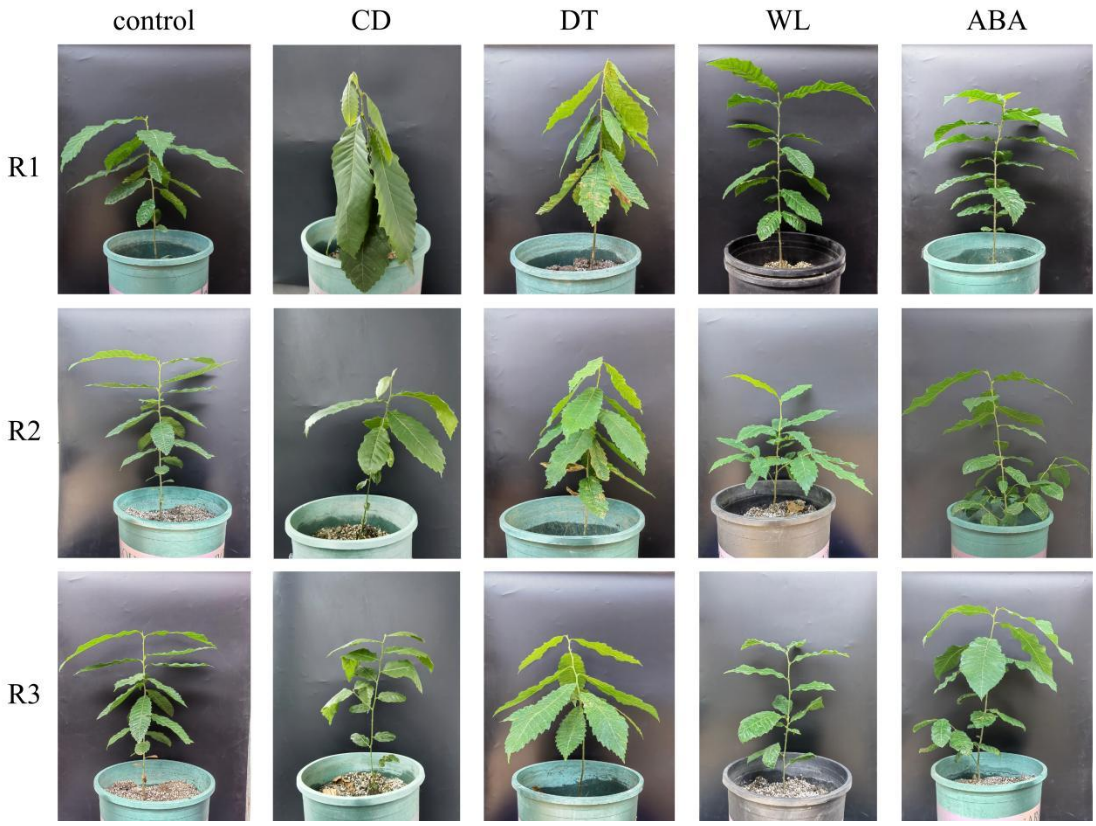

Figure S6. 'Yanbao' chestnut seedlings used for RNA-seq.

CD: cold (low temperature); DT: drought; WL: waterlogging; ABA: exogenous ABA.

R1, R2, and R3 represent three biological replicates, respectively.

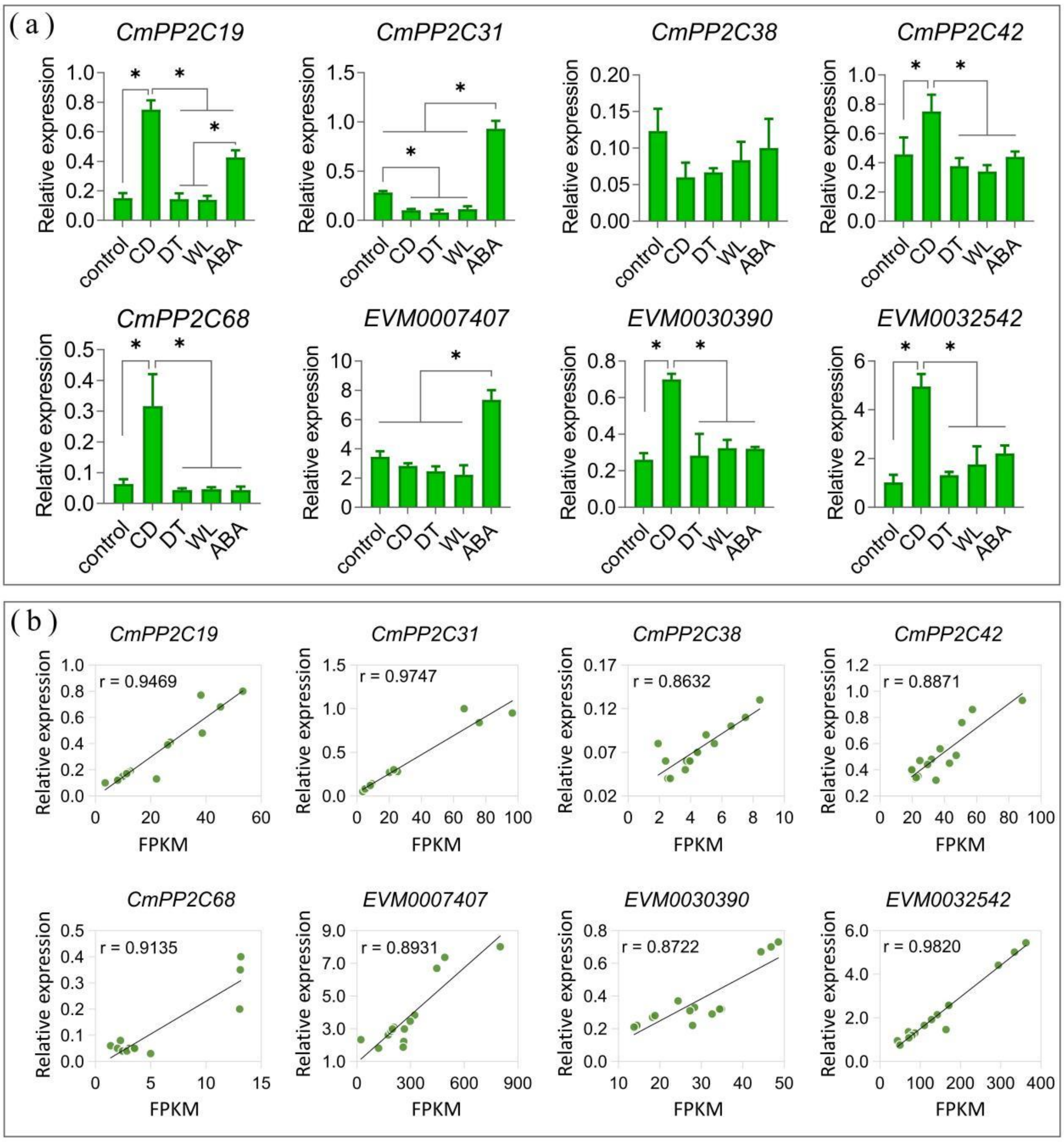

Figure S7. Relative expression patterns of five *CmPP2C*s and three transcription factor genes under different treatments determined by RT-qPCR (a), and correlation analysis with FPKM values (b). * indicates significant differences at P < 0.05, as determined using Student's t-test. The *r* value represents the Pearson correlation coefficient.

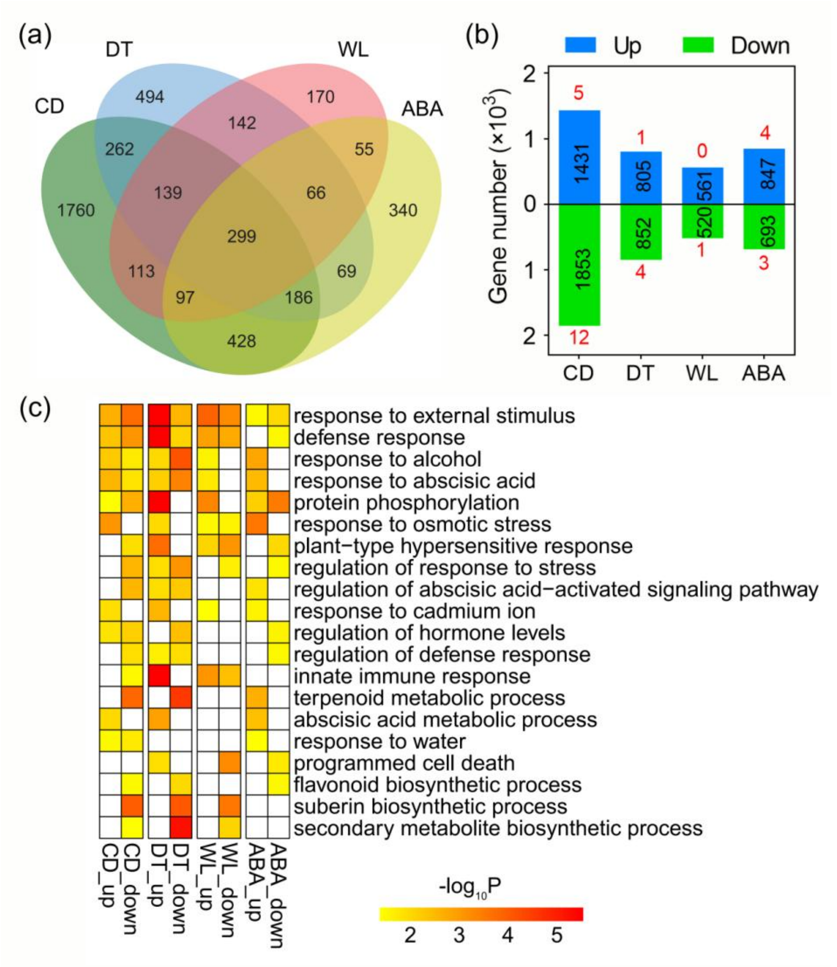

Figure S8. Identification and enrichment analysis of DEGs.

(a) The Venn diagram shows the number of DEGs under four treatments and their cross relationships. The DEGs in the CD, DT, WL, and ABA treatments were identified by comparing them with the control. CD: cold (low temperature); DT: drought; WL: waterlogging; ABA: exogenous ABA. (b) The number of DEGs in the four treatments. Red represents the number of *CmPP2C*s among the DEGs. (c) Enriched GO terms by the DEGs from the four treatments.

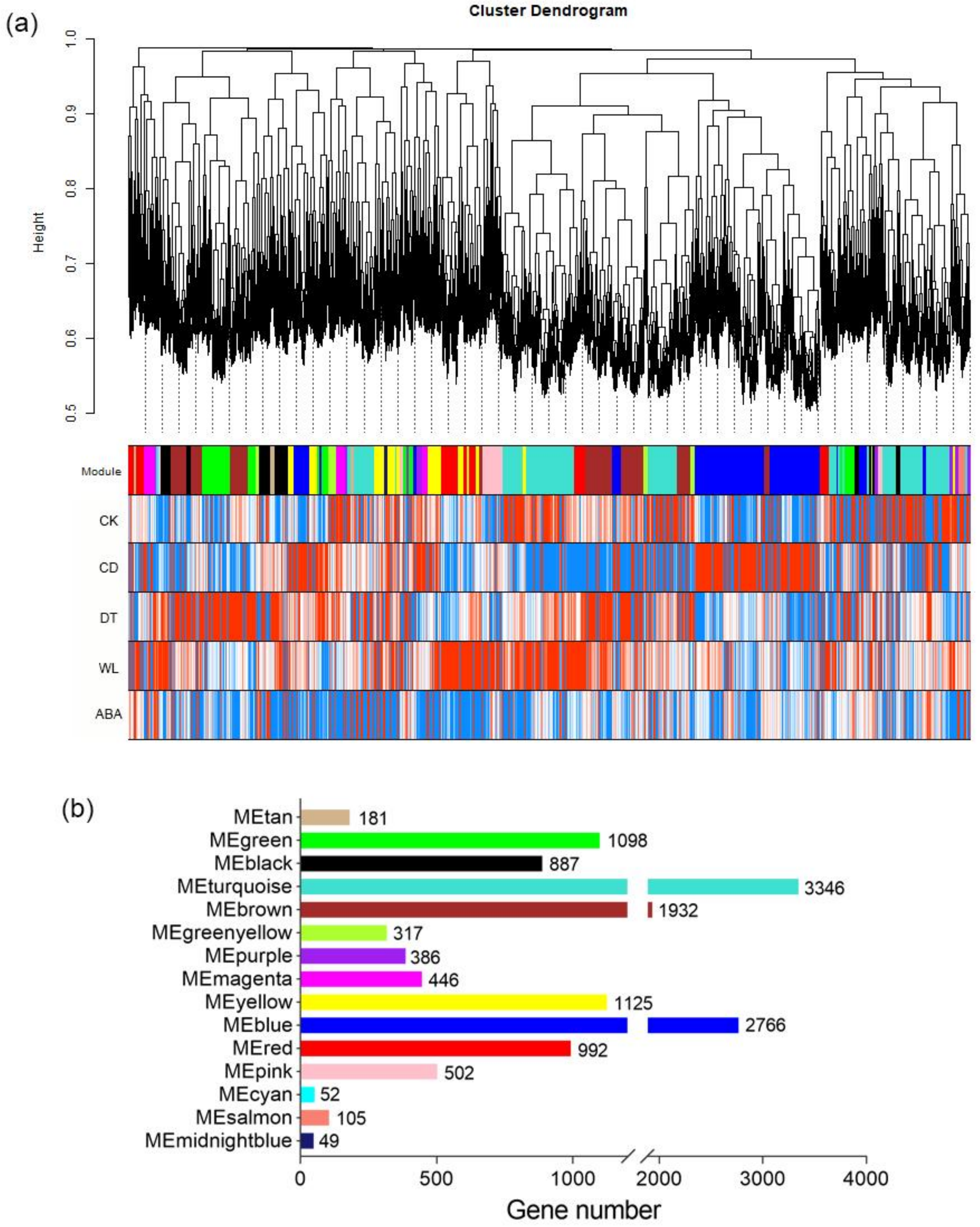

Figure S9. Weighted gene co-expression network analysis of all expressed genes. (a) The clustering dendrogram of all expressed genes, with dissimilarity based on topological overlap, together with assigned module colors. (b) The numbers of genes harbored in each module.

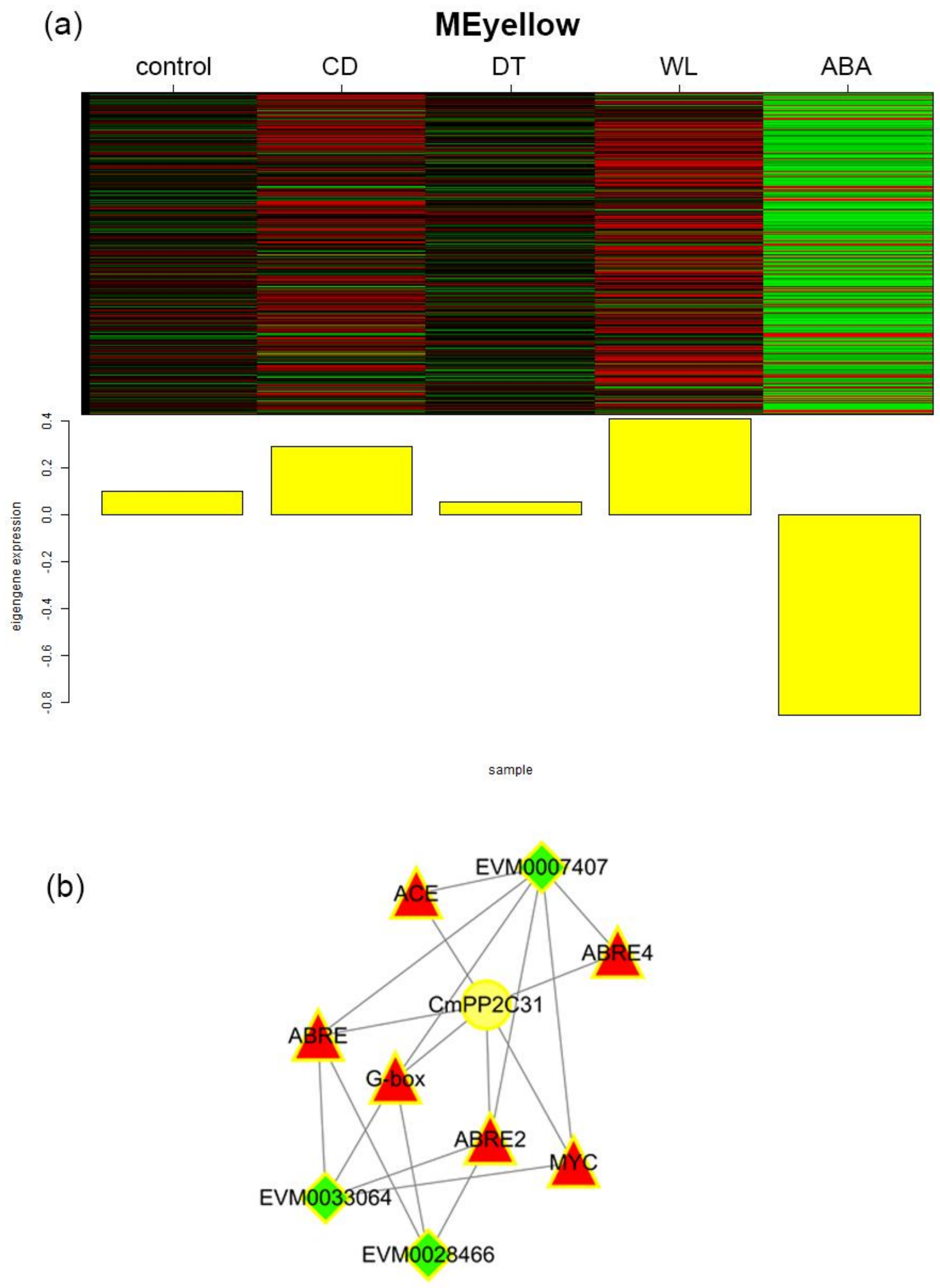

Figure S10. The eigengene expression of MEyellow module (a) and network relationship between *CmPP2C31* and cis-acting elements, as well as between these elements and transcription factors (b). Green diamonds represent transcription factors, yellow circle indicates *CmPP2C31*, red triangles represent cis-acting elements.

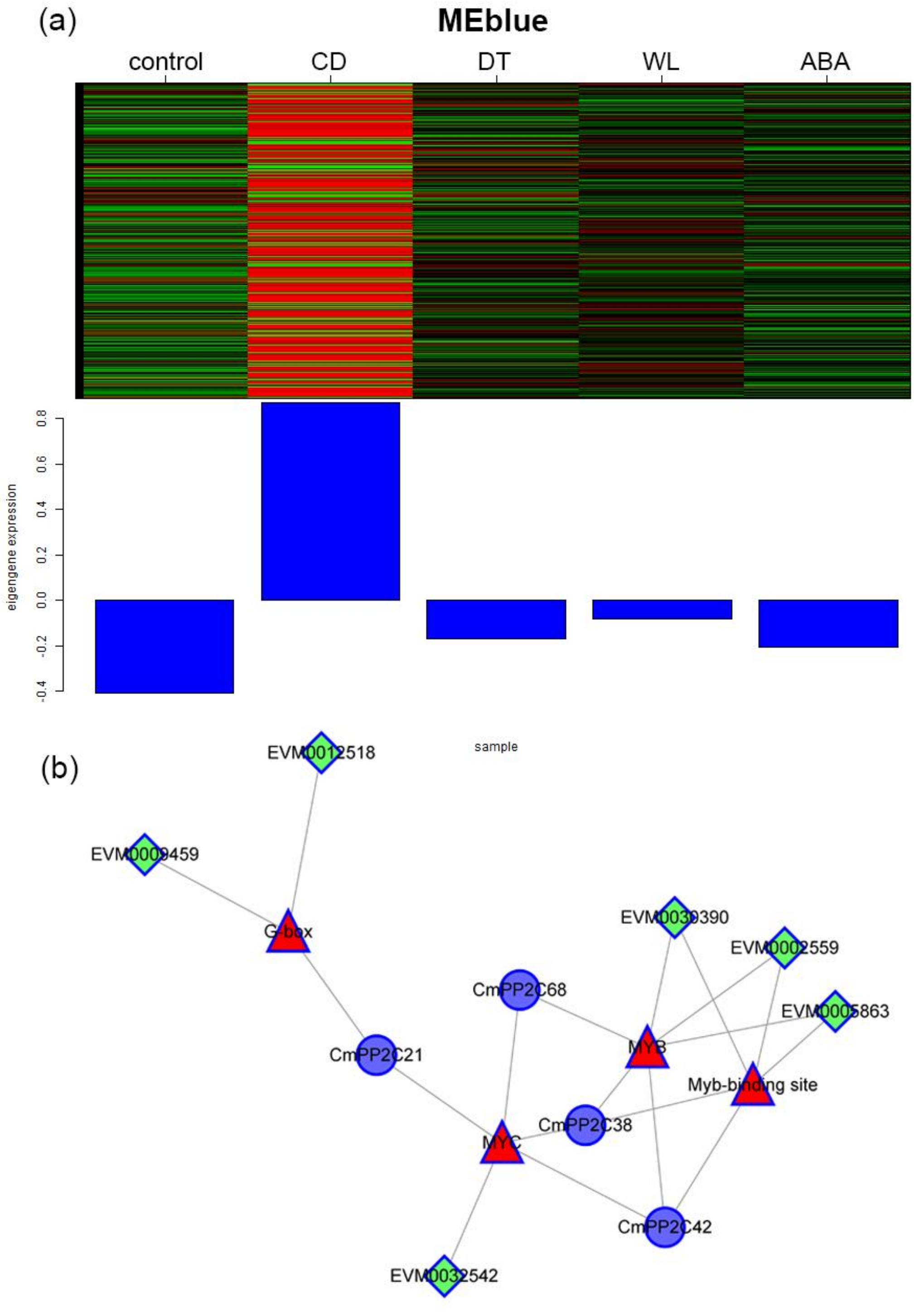

Figure S11. The eigengene expression of MEblue module (a) and network relationship between *CmPP2C*s and cis-acting elements, as well as between these elements and transcription factors (b). Green diamonds represent transcription factors, blue circles indicates *CmPP2C*s, red triangles represent cis-acting elements.

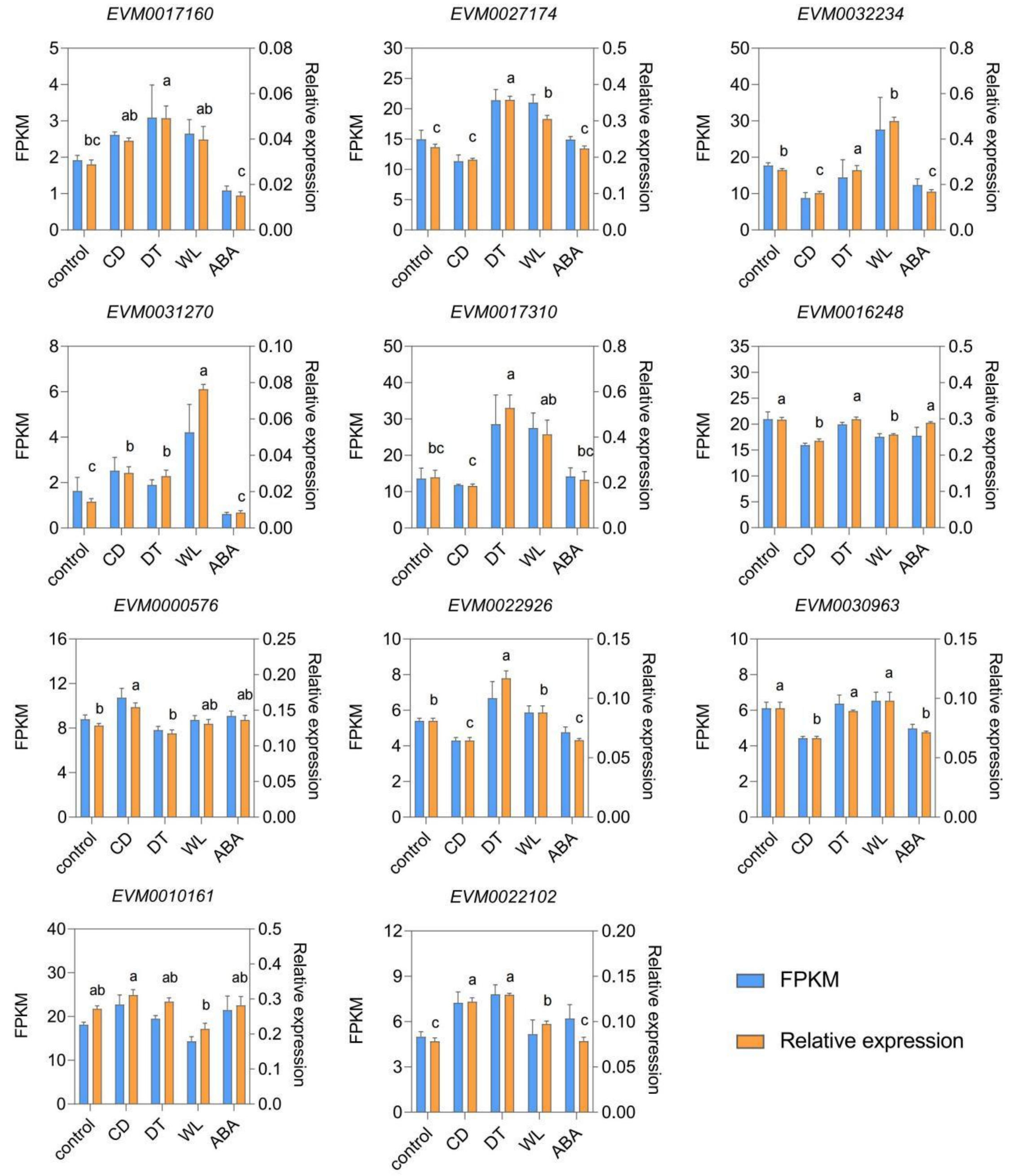

Figure S12. FPKMs and relative expressions of *MYB* (*EVM0017160*, *EVM0027174*, *EVM0032234*, *EVM0031270*, *EVM0017310*), *BRM* (*EVM0016248*), *HDA* (*EVM0000576*, *EVM0022926*, *EVM0030963*), and *CBF* (*EVM0010161*, *EVM0022102*) genes. The letters at the top of the bars represent significance analysis ($P < 0.05$) based on relative expression. There is a significant difference between two samples without the same letter.

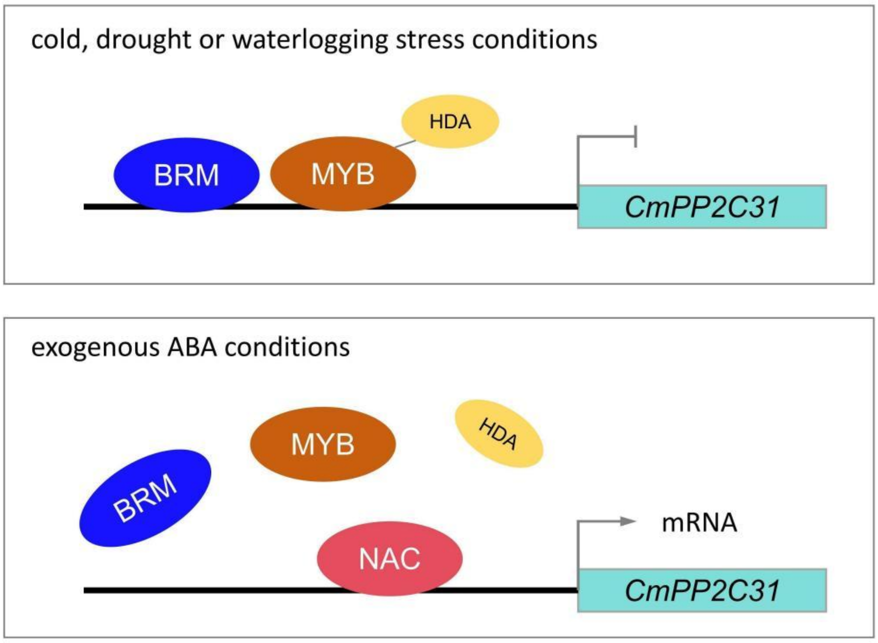

Figure S13. Hypothetical transcriptional regulation mode of *CmPP2C31* gene (refer to Nguyen et al., 2019).
