# Supplementary material for: Identification and characterization of CmPP2C31 playing a positive role in the abiotic stress resistance of Chinese chestnut via an integrated strategy
Source: Front Plant Sci. 2024 Dec 13;15:1491269. doi: 10.3389/fpls.2024.1491269 (PMC11671270; doi:10.3389/fpls.2024.1491269)
Supplement: Supplementary file 4 [file Table2.pdf]

Table S3. Information of 10 conservative motifs of 68 CmPP2C proteins

Motif	Length (aa)	Logo of motif
1	46	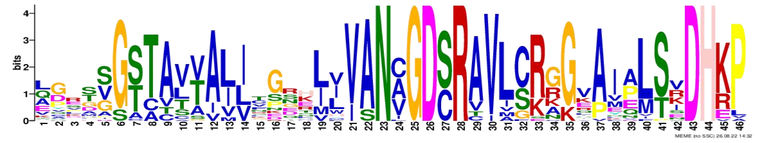
2	17	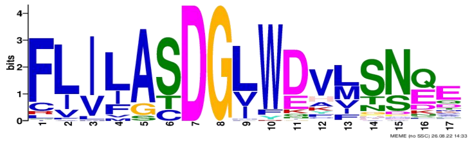
3	21	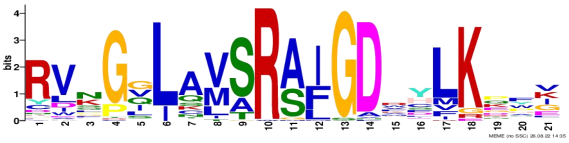
4	28	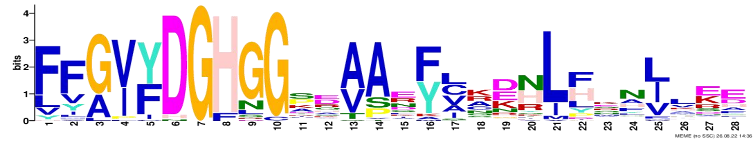
5	29	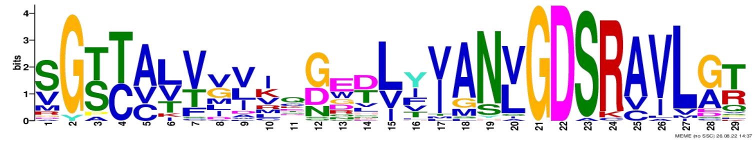
6	57	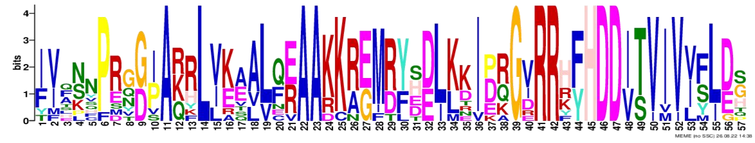
7	29	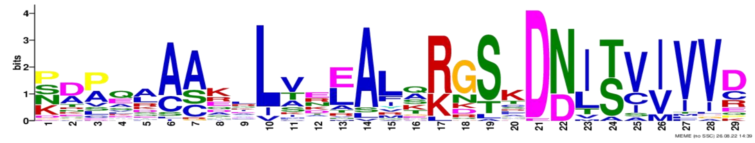
8	15	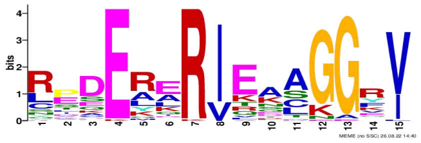
9	41	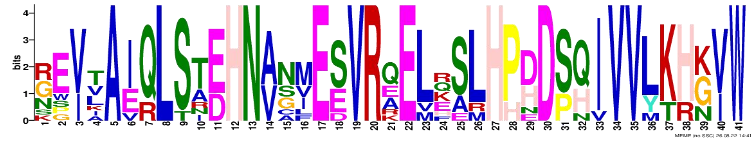
10	25	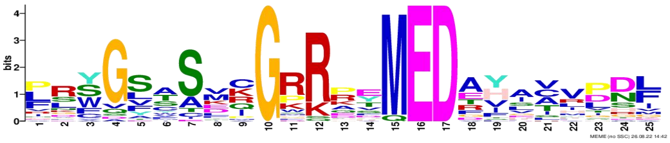
